# Regenerating zebrafish fin epigenome is characterized by stable lineage-specific DNA methylation and dynamic chromatin accessibility

**DOI:** 10.1186/s13059-020-1948-0

**Published:** 2020-02-27

**Authors:** Hyung Joo Lee, Yiran Hou, Yujie Chen, Zea Z. Dailey, Aiyana Riddihough, Hyo Sik Jang, Ting Wang, Stephen L. Johnson

**Affiliations:** 1 Department of Genetics, Washington University School of Medicine, St. Louis, MO 63110 USA; 2 Edison Family Center for Genome Sciences and Systems Biology, Washington University School of Medicine, St. Louis, MO 63110 USA; 3 McDonnell Genome Institute, Washington University School of Medicine, St. Louis, MO 63108 USA

**Keywords:** Regeneration, DNA methylation, Fate restriction, Zebrafish, Fin, Osteoblast, Chromatin accessibility

## Abstract

**Background:**

Zebrafish can faithfully regenerate injured fins through the formation of a blastema, a mass of proliferative cells that can grow and develop into the lost body part. After amputation, various cell types contribute to blastema formation, where each cell type retains fate restriction and exclusively contributes to regeneration of its own lineage. Epigenetic changes that are associated with lineage restriction during regeneration remain underexplored.

**Results:**

We produce epigenome maps, including DNA methylation and chromatin accessibility, as well as transcriptomes, of osteoblasts and other cells in uninjured and regenerating fins. This effort reveals regeneration as a process of highly dynamic and orchestrated transcriptomic and chromatin accessibility changes, coupled with stably maintained lineage-specific DNA methylation. The epigenetic signatures also reveal many novel regeneration-specific enhancers, which are experimentally validated. Regulatory networks important for regeneration are constructed through integrative analysis of the epigenome map, and a knockout of a predicted upstream regulator disrupts normal regeneration, validating our prediction.

**Conclusion:**

Our study shows that lineage-specific DNA methylation signatures are stably maintained during regeneration, and regeneration enhancers are preset as hypomethylated before injury. In contrast, chromatin accessibility is dynamically changed during regeneration. Many enhancers driving regeneration gene expression as well as upstream regulators of regeneration are identified and validated through integrative epigenome analysis.

## Background

While adult mammals have a limited capacity to regenerate a lost body part, salamanders and fish exhibit outstanding regeneration ability in many body parts including internal organs and appendages [1]. The zebrafish caudal fin has been an excellent model for studying vertebrate tissue regeneration [2, 3]. After amputation, the zebrafish caudal fin robustly regenerates via formation of blastema. A blastema is a mass of proliferative and morphologically homogeneous cells that have the capability to grow and develop into a lost body part. Historically, the blastema was thought to be a homogenous population of multipotent cells capable of differentiating into different cell types [4, 5]. But it has been shown that the zebrafish blastema forms from adult stump cells and is composed of lineage-restricted cells that retain memory of their cell-type origin [6–9]. Similar fate restriction of regenerating cells is also observed in salamander limbs [10] and mouse digit tips [11, 12], suggesting that a regeneration mechanism through lineage-restricted cells is evolutionarily conserved.

Zebrafish caudal fin consists of 16–18 bony rays and soft inter-ray tissue. The key regenerative units are these bony rays, which are segmented and lined by osteoblasts, the specialized bone-producing cells in vertebrate skeleton. Following amputation, osteoblasts undergo dedifferentiation and proliferation to form part of blastema [7, 8]. These dedifferentiated osteoblasts in the blastema only contribute to osteoblast regeneration while other lineage-restricted cells regenerate their original cell type. The underlying mechanism of how lineage restriction during regeneration is regulated and defined has not been understood.

DNA methylation plays a crucial role in establishing and maintaining cell identity in normal vertebrate development. Different developmental stages have distinct DNA methylation patterns, which help shape developmental decisions [13, 14]. In embryogenesis, DNA methylation is dynamically reprogrammed to ensure totipotency [15–17]. Additionally, developmental enhancers become demethylated in a lineage-specific manner [18–21]. Terminally differentiated cells and tissues have their own specific DNA methylation signatures [22, 23]. Thus, it is proposed that DNA methylation defines and stabilizes cellular identity and developmental state.

With this rationale, we reason that the DNA methylation signatures defining cell types could be tightly linked to the restriction of cell fates during regeneration. Each population of fate-restricted cells in the blastema is from various terminally differentiated cells in the stump. These fate-restricted cells keep a memory of their cell-type origin, but their morphology, function, and gene expression profiles are distinct from their cell-type origin. We reasoned that cell-type-specific DNA methylation signatures could contribute to restricting cell fate during regeneration.

Here, we produced and analyzed comprehensive DNA methylome, transcriptome, and chromatin accessibility maps of regenerating osteoblasts over the course of zebrafish fin regeneration. We found that osteoblast-specific DNA methylation signatures are retained during regeneration, suggesting that DNA methylation does not specify different states of regeneration, but instead, serves as a carrier of cell fate memory as cells regenerate. Thus, the highly dynamic regeneration gene expression patterns did not correlate with DNA methylome dynamics; rather, they strongly correlated with chromatin accessibility dynamics in a cell-type-specific manner. Integrating transcriptomes and chromatin accessibility maps, we identified thousands of novel regeneration enhancer elements and validated the enhancer activity of a few dozen candidates. Interestingly, these regeneration enhancers were marked by hypomethylation but closed chromatin conformation in uninjured tissues. Finally, we constructed gene regulatory networks important for fin regeneration by utilizing information gathered from epigenetic and transcriptomic dynamics. Our efforts uncovered that the knockout of the predicted upstream transcription factor Fra1 resulted in disruption of normal regeneration, validating our prediction.

## Results

### DNA methylation of zebrafish fin regenerates is stably maintained during regeneration

To understand how DNA methylation changes during zebrafish fin regeneration, we collected samples from regenerating fins at three different time points (1 day post-amputation (dpa), 2 dpa, and 4 dpa; Additional file 1: Figure S1a) together with uninjured zebrafish fin (0 dpa). We generated whole genome bisulfite sequencing (WGBS) libraries from these samples with a decent amount of CpG coverage (average 11.2× coverage and 62.4% of CpGs covered ≥ 5×; Additional file 1: Figure S1b; Additional file 2: Table S1). The genome-wide CpG methylation was maintained at levels as high as around 80% during fin regeneration (Additional file 1: Figure S1c). Around 70% of CpGs were highly methylated, whereas a small proportion of CpGs were intermediately methylated or unmethylated, exhibiting typical bimodal distribution of CpG methylation (Additional file 1: Figure S1d, e). The unmethylated CpGs were mostly found in lowly methylated gene promoters (Additional file 1: Figure S1e), as previously described for zebrafish embryos [16, 17, 20] and other vertebrates [24, 25]. The average DNA methylation levels around genic regions were also consistent across different time points (Additional file 1: Figure S1e). These results suggest that a global change of DNA methylation, typically referred to as reprogramming, is not accompanied with a regeneration process. It is noteworthy that the samples we collected included mixed populations of blastema and wound epidermis from regenerating fins. Thus, we cannot completely exclude the possibility that global DNA methylation changes occur only in the blastema as previously reported using immunohistochemistry [26].

To identify local genomic regions with DNA methylation changes across different time points during regeneration, we searched for differentially methylated regions (DMRs) by using the statistical method DSS [27, 28]. To our surprise, the numbers of DMRs between two different time points were very low and were not larger than the numbers of false positive DMRs defined as those predicted between two biological replicates (Additional file 1: Figure S1f). Thus, the majority of, if not all, DMRs predicted between two time points were likely false positives. This holds true under different statistical cutoffs (Additional file 1: Figure S1g) or using a different computational tool (Additional file 1: Figure S1h). These results suggest that overall DNA methylation levels are stably maintained at both global and local levels during fin regeneration.

### Lineage-specific DNA methylation signatures are stably maintained during fin regeneration

**Fig. 1:**
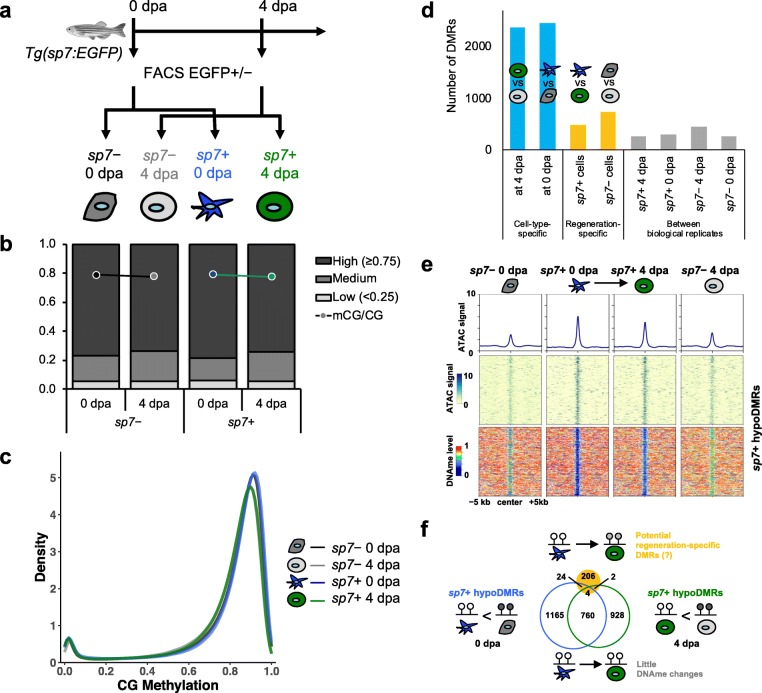
Lineage-specific DNA methylation signatures are stably maintained during fin regeneration. **a** Experimental scheme of sorting *sp7*+ and *sp7*− cells from uninjured and regenerating zebrafish fin by using FACS. **b** Global CpG methylation levels (mCG/CG) and fraction of total CpGs with low (< 25%), medium (≥ 25% and < 75%), and high (≥ 75%) methylation levels of *sp7*+ and *sp7*− cells during zebrafish fin regeneration. **c** Distribution of genome-wide CpG methylation levels of each cell type. Bimodal distribution of two CpG populations at high and low methylation levels is observed. **d** Number of DMRs identified between two biological replicates (gray bars), between two different time points in the same cell type (regeneration-specific, yellow bars) or between two different cell types at the same time point (cell-type-specific, blue bars). **e** ATAC-seq signals (top) and DNA methylation levels (bottom) over 10-kb regions centered on a total of 2883 *sp7*+ cell-specific hypoDMRs. Average ATAC-seq signals were plotted on top of each heatmap (line plots). **f** Venn diagram of *sp7*+ cell-specific hypoDMRs (blue and green circles for 0 dpa and 4 dpa, respectively) intersecting with potential regeneration-specific DMRs in *sp7*+ cells (yellow filled circle). Only 30 (1.0%) of *sp7*+ cell-specific hypoDMRs were predicted as potential regeneration-specific DMRs

Having established that the overall DNA methylation levels are maintained in regenerating fin tissues, we next asked whether this pattern holds true in specific cell types, especially in cells that form a blastema. To this end, we set out to define the epigenomic and transcriptional signatures of regenerating osteoblasts. We took advantage of *Tg(sp7:EGFP)* stable lines, which express EGFP in differentiating and mature osteoblasts [29]. We examined a time-course expression of EGFP in regenerating fins and confirmed that EGFP expression was localized to the segmented bony fin rays in uninjured fin and strongly upregulated in the regenerates (Additional file 1: Figure S2a) [7, 29]. We separated *sp7*+ cells from *sp7*− cells by using fluorescence-activated cell sorting (FACS) after dissociating uninjured fins (0 dpa) or fin regenerates (4 dpa) into a single-cell suspension (Fig. 1a; Additional file 1: Figure S2b). To define DNA methylation dynamics, we generated WGBS libraries from *sp7+* and *sp7*− cells from both time points and sequenced to high-depths (average 25.1× coverage and 79.0% of CpGs covered ≥5×; Additional file 1: Figure S2c; Additional file 2: Table S1). Overall, both *sp7*+ and *sp7*− cells were globally highly methylated during regeneration, with average CpG methylation levels of around 78% (Fig. 1b). Genome-wide CpG methylation levels exhibited a typical bimodal distribution with peaks at high (75–100%) and low (0–25%) methylation levels (Fig. 1c). The global DNA methylation patterns over genic regions superimpose each other, suggesting that there is no dramatic global difference in DNA methylation between the two stages (Additional file 1: Figure S2d), consistent with the result from DNA methylation analysis of bulk regenerates.

To identify dynamics of DNA methylation at local genomic regions during the regeneration processes, we again searched for DMRs using DSS [27, 28]. We examined the number of DMRs identified at different *p* value cutoffs. Interestingly, the number of DMRs detected between *sp7*+ cells of 0 dpa and 4 dpa was low and similar to the baseline established by the comparison between biological replicates (Additional file 1: Figure S2e). Thus, the number of discovered regeneration-specific DMRs is similar to the number of DMRs expected by chance, and the estimated false discovery rate is high. These DMRs might reflect biological variation or stochasticity and do not have enough statistical confidence to be determined as regeneration-specific DMRs. This suggests that the small number of regeneration-specific DMRs were most likely false positives. In contrast, the number of cell-type-specific DMRs (*sp7*+ vs. *sp7*−) drastically outnumbered the number of regeneration-specific DMRs (0 dpa vs. 4 dpa) (Fig. 1d; Additional file 1: Figure S2e). To avoid any bias from the DMR calling algorithm, we tested a different algorithm and obtained similar results (Additional file 1: Figure S2f). In total, we identified 2154 and 2029 *sp7*+ cell-specific DMRs in 0 dpa and 4 dpa samples, respectively. In the majority of those DMRs (91% at 0 dpa and 84% at 4 dpa), methylation level was lower in *sp7*+ cells than in *sp7*− cells (Additional file 1: Figure S2 g). Tissue-specific hypomethylated DMRs (hypoDMRs) are a signature of tissue-specific regulatory regions [19, 20, 23, 30]; therefore, these hypoDMRs are likely regions with *sp7*+ cell-specific regulatory activities. To investigate whether *sp7*+ cell-specific hypoDMRs were associated with regulatory activities, we generated ATAC-seq libraries of the same samples (Fig. 1a; Additional file 2: Table S1; see below). Indeed, *sp7*+ cell-specific hypoDMRs exhibited much higher ATAC-seq signals than their neighboring regions in *sp7*+ cells while ATAC-seq signals were absent over these hypoDMRs in *sp7*− cells (Fig. 1e). These results suggest that *sp7*+ cell-specific hypoDMRs are putative regulatory elements specific for osteoblast lineage cells. It is noteworthy that we identified much fewer *sp7*− cell-specific hypoDMRs than *sp7*+ cell-specific hypoDMRs (Additional file 1: Figure S2g). This is likely due to *sp7*− cells representing a heterogeneous cell population of multiple different lineages.

To test whether fate-restricted osteoblasts maintain their lineage-specific DNA methylation signatures during regeneration, we compared DNA methylation levels of *sp7*+ cell-specific hypoDMRs in 0 dpa and 4 dpa samples. The majority (2734 out of 2883, 95%) of *sp7*+ cell-specific hypoDMRs displayed little change in DNA methylation (< 0.25) during regeneration (Additional file 1: Figure S2h, i) and only 30 (1.0%) DMRs were predicted to be associated with regeneration (Fig. 1e, f). In addition, the vast majority (> 99%) of all the lowly methylated regions in *sp7*+ cells displayed little change in DNA methylation during regeneration (Additional file 1: Figure S2j). Altogether, these results suggest that lineage-specific DNA methylation signatures undergo very few, if any, changes during regeneration. These results suggest that restricted cell fate during regeneration is tightly associated with lineage-specific DNA methylation signature.

### Regeneration-specific genes are activated independent of DNA methylation changes

**Fig. 2:**
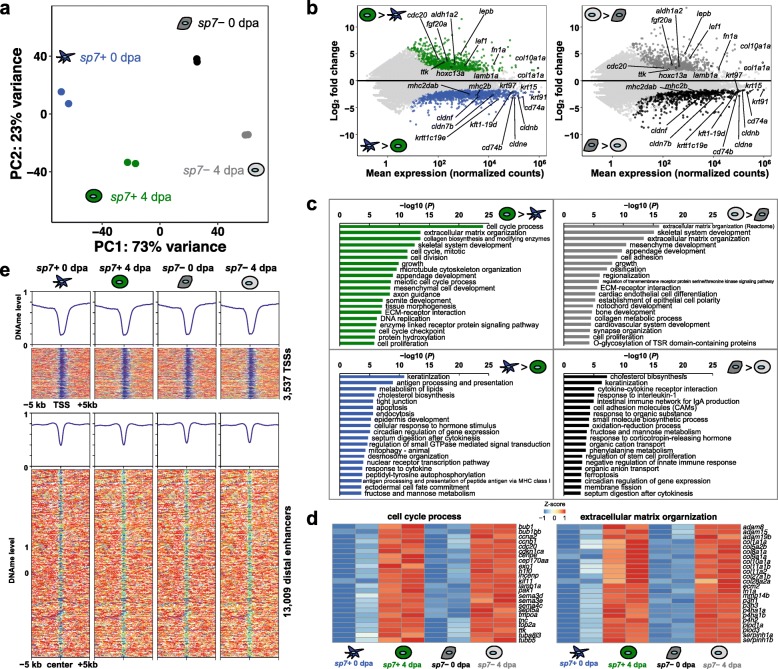
Regeneration-specific genes are activated independent of DNA methylation changes. **a** Principal component analysis on the transcriptomes of *sp7*+ and *sp7*− cells at 0 dpa uninjured fin and 4 dpa blastema. **b** MA plots for differentially expressed genes during fin regeneration in *sp7*+ and *sp7*− cells. Each dot represents log-transformed individual gene expression change. Green and dark gray dots represent statistically significantly upregulated genes during regeneration in *sp7*+ and *sp7*− cells, respectively (log_2_(FC) > 1 and FDR < 0.05). Blue and black dots represent genes statistically significantly downregulated genes during regeneration in *sp7*+ and *sp7*− cells, respectively (log_2_(FC) < − 1 and FDR < 0.05). Light gray dots represent genes with no significant changes. **c** Gene ontology (GO) terms associated with significantly differentially expressed genes. **d** Examples of expression pattern of upregulated genes during regeneration that fall within the top GO terms. **e** DNA methylation levels over 10 kb around the promoters and putative distal enhancers of the significantly differentially expressed genes during regeneration

How is the maintenance of DNA methylation signatures related to transcriptomic dynamics in the process of regeneration? To investigate this question, we generated high-quality RNA-seq of *sp7*+ and *sp7*− cells from 0 dpa uninjured fins and 4 dpa fin regenerates (Additional file 1: Figure S3a, b; Additional file 2: Table S1). The principal component analysis of the transcriptomes effectively separated samples according to their biological states (Fig. 2a). The first principal component (PC1) separated *sp7*+ cells from *sp7*− cells, while PC2 separated 4 dpa blastema from uninjured fins (0 dpa), indicating that regenerating cells underwent enormous transcriptional changes during fin regeneration. A total of 2914 and 1794 genes displayed > 2-fold differential transcript abundance through fin regeneration in *sp7*+ and *sp7*− cells, respectively (FDR < 0.05), with 1125 common between *sp7*+ and *sp7*− cells (Fig. 2b; Additional file 1: Figure S3c; Additional file 3: Table S2).

Genes upregulated during regeneration in both *sp7*+ and *sp7*− cells included many previously described fin regeneration genes, such as *fgf20a* [31], *hoxc13c* [32], and *lepb* [33], suggesting the existence of a common genetic program for regeneration. The upregulated genes during regeneration were enriched for biological functions including cell cycle process, extracellular matrix organization, and appendage development (Fig. 2c), consistent with cellular processes characteristic of fin regeneration. We observed that upregulation of genes was associated with cell cycle (*cdc20*, *ccnb1*, *exo1*, and *cenpe*) and appendage development (*and1*, *and2*, *bmp1a*, *lef1*, and *dlx5a*), suggesting that regenerating cells actively proliferate and reinitiate developmental programs (Fig. 2d; Additional file 1: Figure S3d). We also observed that downregulation of genes during regeneration was associated with keratinization (*col17a1a*, *krt15*, *krt91*, and *krt97*), cholesterol biosynthesis (*acot11a*, *asct3a*, *apodb*, and *cyp51*), and tight junction (*cldn7b*, *cldnb*, *cldne*, and *pard6a*) (Additional file 1: Figure S3d). Downregulation of these genes is consistent with extensive disorganization of tissues during blastema formation and growth.

Although genome-wide DMR prediction did not return any genomic regions with significant DNA methylation changes, we wanted to examine DNA methylation dynamics of regulatory elements associated with genes whose expression changed during regeneration to potentially identify any elements that escaped genome-wide DMR detection. Consistent with our genome-wide DNA methylation analysis (Fig. 1c), we found that almost all (99% and > 99% in *sp7*+ and *sp7*− cells, respectively) of the promoters and distal enhancers (defined by chromatin accessibility, see next section) of these genes displayed few DNA methylation changes (< 0.25) during regeneration (Fig. 2e; Additional file 1: Figure S3e). Only 5 distal enhancers showed significant DNA methylation changes by DSS in *sp7*− cells, which can be due to the different cellular heterogeneity at 0 dpa and 4 dpa. This result suggests that DNA methylation is not a major regulator of gene transcription in the regeneration program. Therefore, epigenetic regulation mechanisms other than DNA methylation should play a role to regulate regeneration-specific gene transcription.

### Regeneration-specific gene activation is associated with increasing chromatin accessibility

Accessible chromatin has been described as a general feature for regulatory elements including promoters and enhancers [34, 35]. Therefore, we set out to test the hypothesis that chromatin accessibility is the key in regulating regeneration-specific gene transcription. To this end, we generated ATAC-seq libraries from the same sorted cells (Fig. 1a; Additional file 2: Table S1). We identified a total of 111,941 highly reproducible accessible chromatin regions in *sp7*+/− cells of the uninjured and regenerating fins. PCA analysis separated *sp7*+ cells from *sp7*− cells along PC1, while 4 dpa fin regenerates and uninjured fins (0 dpa) were separated along PC2 (Additional file 1: Figure S4a), recapitulating the pattern obtained from gene expression analysis. We found that 26% of ATAC peaks were in promoters (2 kb regions around transcription start site (TSS)), while 45% of ATAC peaks were distal (> 10 kb) to TSS (Additional file 1: Figure S4b). The expression levels of genes with accessible chromatin in their promoter were significantly higher than those of genes without accessible chromatin signatures (Additional file 1: Figure S4c), confirming that active promoters were marked with accessible chromatin.

**Fig. 3:**
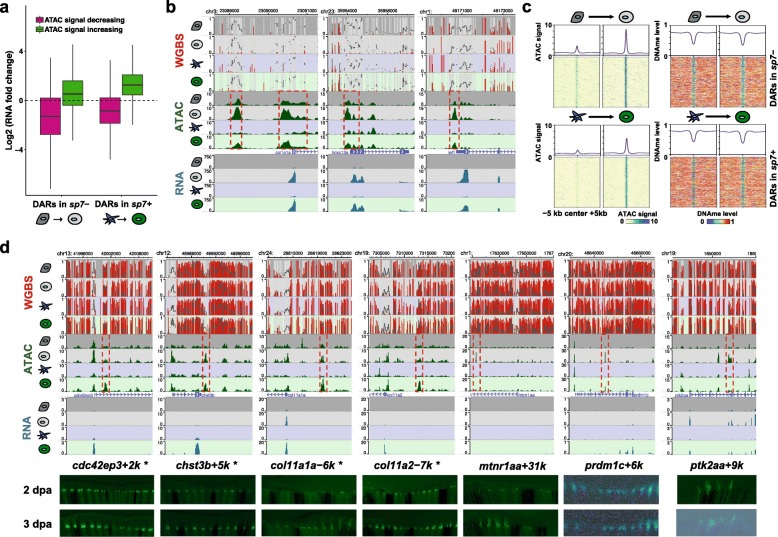
Regeneration-specific gene activation with gain of chromatin accessibility. **a** Expression fold changes for the genes with differentially accessible promoters during regeneration. **b** Epigenome browser views of genes whose activation is concordant with gain of chromatin accessibility of the promoter region (*col1a1a*, *hoxc13a*, and *lef1*). Red dashed boxes highlight ATAC peaks with increasing signals during regeneration. **c** ATAC-seq signals (left) and DNA methylation levels (right) over 10-kb regions centered on DARs with increasing signals in *sp7*+ (top heatmap) and *sp7*− cells (bottom heatmap) during regeneration. Average ATAC-seq signals and DNA methylation levels were plotted on top of each heatmap (line plots). **d** Identified candidates of regeneration-related enhancers and in vivo validations. Epigenome browser views of regeneration enhancers (top) and transgenic zebrafish carrying candidate sequence-driven reporter showing enhancer activities in the regenerating fin (bottom). Red dashed boxes indicate DARs that gained accessibility during regeneration. Asterisks indicate that F_1_ transgenic zebrafish line was established for a given enhancer element

To investigate the dynamics of chromatin accessibility during regeneration, we identified differentially accessible regions (DARs) by using DiffBind [36]. A total of 15,197 and 19,016 peaks displayed > 2-fold differential accessibility during regeneration in *sp7*+ and *sp7*− cells, respectively (FDR < 0.01), with 19% of them in promoter regions (Additional file 1: Figure S4d, e; Additional file 4: Table S3). To determine whether these dynamic chromatin accessibility changes reflect gene expression changes globally, we compared the direction of changes in neighboring gene expression. During regeneration, genes near DARs with increasing ATAC-seq signals tended to also increase their expression levels, while genes near DARs with decreasing ATAC-seq signals showed decreased expression levels (Fig. 3a; Additional file 1: Figure S4f). Similarly, genes that were upregulated or downregulated during regeneration exhibited increased and decreased ATAC-seq signals in their genomic neighborhood, respectively (Additional file 1: Figure S4g). For example, expression activation of *hoxc13a*, *lef1*, and *col1a1a* co-occur with their promoters gaining chromatin accessibility (Fig. 3b). This result indicates that increasing chromatin accessibility of regulatory elements was positively correlated with increased expression of target genes during regeneration.

Previous studies suggested that DNA demethylation of active cis-regulatory elements is often correlated with gain of chromatin accessibility or active enhancer histone marks [21, 37]. However, in our case, consistent with the above results, DNA methylation levels of DARs were mostly stable during regeneration (99% and 97% DARs with < 0.25 DNA methylation changes in *sp7*+ and *sp7*− cells, respectively, Fig. 3c; Additional file 1: Figure S4h). Only 28 DARs were determined to exhibit significant DNA methylation changes by DSS in *sp7*− cells, which might be exceptions due to the different cellular heterogeneity at different stages of regeneration. These DARs have distinct genetic and epigenetic characteristics from the previously determined zebrafish developmental enhancers [21, 38] (Additional file 1: Figure S5). Intriguingly, the majority of the DARs with increasing accessibility were lowly or intermediately methylated (< 0.6) in uninjured cells, pre-regeneration (84% and 85% in *sp7*+ and *sp7*− cells, respectively). In terminally differentiated cells, many developmental genes were repressed by epigenetic mechanism, such as DNA methylation over their cis-regulatory elements. DNA methylation status of the limb developmental enhancer is negatively correlated with the regenerative potential of limb in different developmental stages of frogs [39]. Low levels of DNA methylation over the DARs associated with regeneration genes in uninjured differentiated cells support a model that, in animals with high regeneration abilities, differentiated cells maintain a permissive epigenetic state (lowly methylated, yet low chromatin accessibility) over regulatory elements that are important for regeneration, thus might allow for rapid gene activation when needed for regeneration [40].

To test whether the lowly methylated regions that gain accessibility during regeneration could serve as regeneration-specific enhancers, we selected multiple putative regeneration enhancers located far from gene promoters (> 2 kb, up to 138 kb) and tested their enhancer activities in vivo in the regenerating fin (Table 1). Putative regeneration enhancers were cloned into a minimal promoter-driven GFP reporter cassette [41] (Additional file 2: Table S4). We generated transgenic zebrafish carrying these cassettes via Tol2 transposase system and monitored their reporter activities in the regenerating fin. Regeneration enhancers identified in *sp7*+ cells drove GFP expression along the regenerating fin rays, while *sp7*− regeneration enhancers gave different patterns of GFP expression in the regenerates (Fig. 3d; Additional file 1: Figure S4i). In total, 18 out of 25 (72%) candidates displayed enhancer activities in regenerating fin while no activity was observed from 9 negative controls (Table 1). These results strongly support that the genomic regions which gain chromatin accessibility during regeneration were regeneration-specific enhancers and were responsible for driving gene expression during fin regeneration.

**Table 1:** List of regeneration enhancers tested

Element name	DAR in *sp7*+ cells	DAR in *sp7−* cells	EGFP expression
*bmp2a+43k*	Yes	Yes	–
*chst3b+5k*	Yes	Yes	+
*cygb1−13k*	Yes	Yes	+
*dnajc17−138k*	Yes	Yes	+
*lef1−2k*	Yes	Yes	–
*pdgfab−8k*	Yes	Yes	+
*prdm1c+6k*	Yes	Yes	+
*runx1−102k*	Yes	Yes	–
*cdc42ep3+2k*	Yes	No	+
*col11a1a−6k*	Yes	No	+
*col11a2−7k*	Yes	No	+
*fam102aa+11k*	Yes	No	+
*fbln1+17k*	Yes	No	+
*mef2aa+2k*	Yes	No	+
*mitd1−6k*	Yes	No	–
*prdm5+120k*	Yes	No	+
*rmb7−120k*	Yes	No	–
*swap70b+3k*	Yes	No	+
*fhod3b+138k*	No	Yes	+
*frmd4a+87k*	No	Yes	+
*grip2a−23k*	No	Yes	+
*igflr1−2k*	No	Yes	–
*mtnr1aa+31k*	No	Yes	+
*ptk2aa+9k*	No	Yes	+
*rasl11a−13k*	No	Yes	–
*atp5g3a−14k*	No	No	–
*lmbr1+11k (ZRS)*	No	No	–
*lnpa+56k*	No	No	–
*six3b−102k*	No	No	–
*six3b−104k*	No	No	–
*six3b−36k*	No	No	–
*six3b−39k*	No	No	–
*wnt3−11k*	No	No	–
*zgc:173726−2kb*	No	No	–

Taken together, these results suggest that gene expression changes during regeneration were not regulated by DNA methylation but rather associated with changes of chromatin accessibility. These putative regulatory enhancers for the regeneration were marked with low DNA methylation at 0 dpa, potentially allowing rapid regeneration responses upon injury.

### Construction of gene regulatory networks identifies upstream factors for fin regeneration

**Fig. 4:**
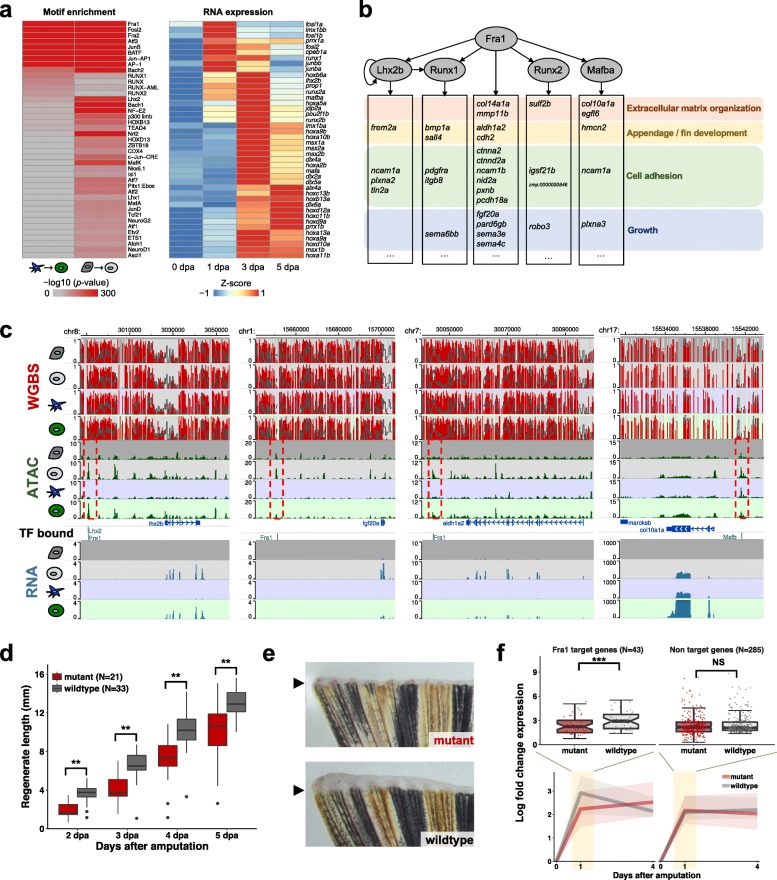
Gene regulatory networks identify upstream factors for fin regeneration. **a** Heatmaps showing the enriched TF binding motifs in DARs that gained accessibility in *sp7*+ and *sp7*− cells during fin regeneration (left) and of RNA expression of the corresponding TF genes (right). Motifs were sorted by binomial *p* value of enrichment in *sp7*+ cells. TF genes were clustered by their expression levels. **b** Putative gene regulatory network of the fin regeneration. The gray ovals are TFs whose motifs were highly enriched in DARs that gained accessibility during regeneration. The genes in the bottom boxes are the target genes of Fra1 and/or other TFs, identified by the footprint analysis. These target genes have biological functions relevant during fin regeneration as shown on the right. **c** Example Epigenome Browser views of Fra1, Lhx2, or Mafb motifs found in DARs, with their nearby target genes. Red dashed boxes indicate DARs that gained accessibility during regeneration. Motifs predicted to be bound by TFs were shown. **d** Boxplot showing fin regenerate lengths as a function of the time after amputation. Mutant (*fosl1a*^*tw4/tw4*^) zebrafish (red) showed delayed regeneration after fin amputation compared to their wildtype littermates (gray). **p < 0.01; Mann–Whitney *U* test. **e** Representative pictures of the fin regenerate from the mutant (*fosl1a*^*tw4/tw4*^) and their wildtype (*fosl1a*^*+/+*^) littermates at 2 dpa. Arrowhead, amputation plane. **f** RNA expression levels of the Fra1 target genes and non-target genes in the mutant (*fosl1a*^*tw1/tw1*^) and their wildtype littermates. The predicted target genes of Fra1 were not upregulated as highly in mutant fish as in wildtype at 1 dpa (left), consistent with the delayed regeneration phenotype. Upregulated genes that were not Fra1 targets showed statistically no difference in the level of gene expression changes (right). Top boxplots show gene expression fold changes at 1 dpa. Bottom line plots show median gene expression fold changes across genes (line) in the time course. Shaded areas represent 25% and 75% quantiles. ***p < 0.001; Wilcoxon signed-rank test

Having established that DARs were active cis-regulatory elements regulating regeneration, we sought to link upstream transcription factors (TFs) to their downstream target genes. We first investigated the TF binding motifs enriched in DARs that gained accessibility during regeneration. We identified binding motifs of many important developmental TFs, including AP1 family and Runx family, that were highly enriched in these DARs (Fig. 4a; Additional file 1: Figure S6a). Many of these TFs were upregulated during fin regeneration as early as 1 dpa (Fig. 4a; H.J.L., C. Higdon, and S.L.J, unpublished data), suggesting that these TFs might be activated prior to their downstream regeneration genes. We used footprint analysis of the TFs to infer TF binding at regeneration-specific DARs and connected TFs to putative target genes (Additional file 1: Figure S6b). We constructed a putative gene regulatory network of fin regeneration by examining regulatory interactions among the TFs and their downstream target genes (Fig. 4b, c) [20]. We identified Fra1 (gene name: *fosl1a*), whose motif enrichment was top ranked, as a putative upstream transcription factor for fin regeneration (Fig. 4a, b; Additional file 1: Figure S6a). It has been shown that Fra1 knockout mice develop osteopenia, a low bone mass disease, indicating that Fra1 functions to regulate bone mass in mice [42]. Many downstream genes predicted to be targeted by Fra1 were linked to biological functions enriched in the upregulated genes during fin regeneration, including appendage development and growth (Fig. 4b). The time-course expression profiles of *fosl1a* gene exhibited dramatic activation immediately after amputation, suggesting that Fra1 might act as an early and upstream TF during fin regeneration (Fig. 4a).

Our analysis predicted that removal of the Fra1 transcription factor prior to amputation might impair caudal fin regeneration by insufficient activation of downstream effectors. To test this prediction, we used CRISPR-Cas9 genome editing technology to knock out *fosl1a*, the gene encoding the Fra1 protein (Additional file 1: Figure S6c). The *fosl1a* mutant zebrafish showed a delayed regeneration rate (Fig. 4d, e). This phenotype is replicated in four independent mutant alleles (Additional file 1: Figure S6c-e), confirming that mutation of *fosl1a* was causal for this regeneration defect. The mutant zebrafish did not show any abnormal developmental or morphological phenotypes and were able to fully regrow their regenerates in 3 weeks, suggesting that the delayed regeneration phenotype is limited to the early stage of regeneration. To see whether this limited regeneration defect is due to genetic compensation by any paralogue of *fosl1a* gene [43, 44], we generated *fosl1b* mutant zebrafish (Additional file 1: Figure S6f) and *fosl1a* and *fosl1b* double mutant zebrafish. The *fosl1b* mutant zebrafish showed no differences from wildtype in regeneration rate and *fosl1a* and *fosl1b* double mutant zebrafish are not significantly different from *fosl1a* mutant (Additional file 1: Figure S6f-h), suggesting that *fosl1b* does not affect regeneration rates. We generated RNA-seq on the wildtype and mutant *fols1a* animal to define molecular phenotype of mutant fish (Additional file 2: Table S1). We found that predicted target genes of Fra1 were not as highly upregulated in mutant fish as in wildtype at 1 dpa during regeneration, while non-target genes were upregulated at similar levels in mutants and their wildtype littermates (Fig. 4f; Additional file 1: Figure S6i). The expression levels of Fra1 target genes in mutant fish caught up the levels in wildtype fish at 4 dpa, consistent with delayed regeneration phenotype in mutant fish. Taken together, this data suggests that the Fra1, predicted to be an upstream factor from a reconstructed regulatory network, activates downstream target genes important for fin regeneration.

## Discussion

Epigenetic modifications, including DNA methylation, have been proposed to be the molecular mechanisms that define cell fate by regulating gene expression. Here we used the zebrafish fin regeneration system, in which different lineages of cells maintain their cell identity during the process, to investigate potential roles of DNA methylation in cell fate restriction. We investigated *sp7*+ osteoblasts and *sp7*− cells at two different stages of fin regeneration with multiple epigenomics assays. We observed the dynamic changes and strong correlations of gene expression and chromatin accessibility during regeneration, but we observed very few DNA methylation changes associated with regeneration. Although *sp7* expression does not label all regenerating osteoblasts, the retained DNA methylation signatures specific for *sp7*+ cells during regeneration still provide support to the model that epigenetic memory in DNA methylation might specify and restrict cell fate.

Regenerative potential varies among species. It has been proposed that adult tissues with a robust regeneration capability have permissive chromatin structures around the genes necessary for regeneration, which are also developmental genes [40]. We detected hundreds of genes activated in fin regeneration and identified regulatory elements responsible for those gene expression changes. These cis-regulatory elements gained chromatin accessibility during regeneration. Interestingly, these regeneration-specific elements were in a hypomethylated but lowly accessible state in uninjured cells, and they gained accessibility while maintaining their hypomethylation status during regeneration. Pre-established low methylation state of regeneration regulatory elements could contribute to the rapid response to the injury by allowing permissive chromatin state of those elements. This idea is consistent with the notion that permissive chromatin state in adult tissue determines the regeneration capability [39, 40]. Whether epigenetic modifications other than DNA hypomethylation, such as bivalent histone modification, in these enhancers also contribute to regeneration capability should be further investigated.

Molecular mechanisms of tissue regeneration have been studied mostly in the context of gene expression and function, and much remains to be investigated to decipher the gene regulatory networks involved in regeneration. Recent work has shown the existence of tissue regeneration enhancer elements that trigger regenerative genetic program upon tissue damage in zebrafish [33]. Another study has used H3.3 replacement histone profiling to identify cardiomyocyte regeneration enhancer elements in injured zebrafish heart [45]. These recent studies suggest that regeneration enhancers exist in different types of regenerating tissues. By combining transcriptome and accessible chromatin analysis, we have identified several thousands of putative regeneration enhancers that orchestrate expression of genes important for fin regeneration. Using transgenic zebrafish, we tested and validated activities of dozens of regeneration enhancers. Temporal and spatial patterns of reporter gene expressions induced by those enhancers varied and displayed cell-type specificity, indicating that different cell types might use different regeneration enhancers. Thus, our study provides a new resource of regeneration regulatory elements, as well as transgenic animals with new markers to facilitate investigation of tissue regeneration.

Genome-wide epigenome profiling not only helps detect genomic regulatory elements but also identifies important candidate genes. By constructing the gene regulatory network of fin regeneration, we identified Fra1 as a putative upstream transcription factor in the network. The binding motif of this transcription factor was highly enriched in regeneration regulatory elements, connecting the TF to many target genes activated in fin regeneration. We validated the involvement of this candidate TF by taking advantage of recently emerging genome editing tools coupled with phenotypic and genomic assays. Further investigation will be required to elucidate the complex mechanism of the regeneration process. Rapidly developing tools such as single-cell and fate-tracing technologies [46] will facilitate broadening our understanding of this complex mechanism.

## Conclusion

Here, we construct epigenome and transcriptome maps of osteoblasts in regenerating zebrafish fin and show that lineage-specific DNA methylation signatures are stably maintained during regeneration while chromatin accessibility and gene expression are dynamically regulated. The epigenetic signatures identify novel regeneration enhancers, most of which are preset as hypomethylated before injury. Integrative analysis reconstructs a regulatory network for regeneration and identifies upstream regulator of regeneration. Our study significantly broadens our understanding role of the epigenetics in tissue regeneration.

## Methods

### Zebrafish maintenance and procedures

All zebrafish were used in accordance with the protocols approved by the Washington University Animal Studies Committee (Protocol #20130107). Wildtype TU, AB, *Tg(sp7:EGFP)* [29], and *Tg(hsp70:zCas9; mylz:CFP)* strains were maintained under standard conditions as previously described [47]. Water temperature was maintained at 28.5 °C for animals. Adult zebrafish ranging in age from 2.5 to 12 months were used unless otherwise indicated. Zebrafish caudal fins were amputated at 50% of their original length using razor blades. After fin amputation, zebrafish were returned to 28.5 °C and allowed to regenerate their fin.

### Isolation of *sp7*+ and *sp7*− cells from zebrafish uninjured and regenerating fins

Osteoblast lineage cells were isolated from uninjured fins or 4 dpa blastema of adult transgenic zebrafish expressing EGFP under the control of the sp7 regulatory regions *Tg(sp7:EGFP)* by using FACS.

For 0 dpa uninjured fins, three to four dissected fins were treated with 1 mL of 1× TrypLE Express enzyme (Gibco) at 37 °C for 30 min in a 1.5-mL microcentrifuge tube with gentle agitation. After TrypLE Express treatment, samples were centrifuged at 500×*g* for 3 min at 4 °C and supernatants were discarded. Samples were washed with 1 mL of 1× cold PBS buffer and centrifuged at 500×*g* for 3 min at 4 °C. Supernatants were discarded, and remaining fins were further dissociated into a single-cell suspension with 1 mL of 0.25 mg/mL Liberase DL (Roche) in enzyme-free Cell Dissociation Buffer (Gibco) at 37 °C for 30 min with gentle agitation. Liberase DL contains highly purified Collagenase I and Collagenase II and facilitates cell dissociation from the intact fin tissue.

For the regenerating blastema, caudal fins were allowed to regenerate at 28.5 °C for 4 days. At 4 days post-amputation, regenerating blastema were collected by cutting them along the amputation plane with a razor blade under the microscope. Blastema from four to ten fish were treated with 1 mL of 1× TrypLE Express enzyme (Gibco) at 37 °C for 60 min in a 1.5-mL microcentrifuge tube with gentle agitation to complete a single-cell suspension.

Dissociated single-cell suspension was washed with 1 mL of cold PBS buffer and pelleted by centrifugation at 500×*g* for 3 min at 4 °C. Cells were resuspended in 1 mL cold PBS buffer with 2% fetal calf serum and filtered through a 50-μm sample preparation filter (CellTrics) to remove cell aggregates. The FACS Aria II flow cytometer (BD Biosciences) was used to separate GFP+ and GFP− populations from single-cell suspension.

### RNA and DNA isolation

For RNA extraction, 12 uninjured fins and 34 blastema per replicate were collected from transgenic zebrafish *Tg(sp7:EGFP)*, and *sp7*+ and *sp7*− cell populations were isolated by using FACS as described above. Approximately 1 million cells were sorted for each gate from FACS. The cells were pelleted by centrifugation at 500×*g* for 5 min at 4 °C. The total RNA was extracted by using TRIzol solution (Ambion) according to the manufacturer’s instructions with minor modifications. Briefly, 1 mL TRIzol was added to the cells and incubated for 5 min at room temperature to permit complete dissociation of the nucleoprotein complex. Then 0.2 mL of chloroform was added and mixed well by shaking for homogenization. The samples were incubated for 3 min at room temperature and centrifuged at 12,000×*g* for 15 min at 4 °C. The aqueous phase of the sample was transferred to a new tube, 1 μL of TURBO DNase (Ambion) was added, and the samples were incubated for 30 min at 37 °C. Then 0.5 mL of 100% isopropanol was added to the aqueous phase and incubated overnight at − 20 °C. The total RNA was pelleted by centrifugation at 12,000×*g* for 10 min at 4 °C. The RNA pellet was washed with 1 mL of 75% ethanol and centrifuged at 7500×*g* for 5 min at 4 °C. The air-dried RNA pellet was then resuspended in 20 μL of RNase-free water by incubating at 55 °C for 10 min. The total RNA concentration was measured by using a Qubit fluorometer (Invitrogen).

For DNA extraction, 15 uninjured fins and 60 blastema per replicate were collected from transgenic zebrafish *Tg(sp7:EGFP)* and *sp7*+ and *sp7*− cell populations were isolated by using FACS as described above. Approximately 1 million cells were sorted for each gate from FACS. The cells were lysed in 400 μL of genomic DNA extraction buffer (50 mM Tris, 1 mM EDTA, 0.5% SDS, 1 mg/mL Proteinase K) for 16 h at 55 °C. Lysis was followed by phenol:chloroform:isoamyl alcohol (25:24:1, PCI) extractions and centrifugation. The supernatant was incubated with RNase for 30 min at 37 °C. Another PCI extraction was performed after RNase treatment, followed by one chloroform extraction. DNA was then precipitated by adding 0.1 volumes of 3 M sodium acetate and 2.5 volumes of 100% ethanol. The DNA precipitate was centrifuged at 16,000×*g* for 15 min at 4 °C, and the pellet was washed with 1 mL of 70% ethanol and centrifuged at 16,000×*g* for 5 min at 4 °C. The pellet was then resuspended in 15 μL of Elution Buffer (10 mM Tris-Cl, pH 8.5). DNA concentrations and purity were measured using NanoVue (GE Healthcare Life Sciences) and Qubit fluorometer (Invitrogen).

### WGBS library generation, sequencing and mapping

A total of 99.5 ng of genomic DNA per replicate, together with 0.5 ng of unmethylated lambda DNA (Promega), was treated with bisulfite and cleaned by using the EZ DNA Methylation-Direct Kit (Zymo Research), according to the manufacturer’s instructions. The unmethylated lambda DNA was used to calculate the bisulfite conversion rate. WGBS libraries were generated from bisulfite-treated genomic DNA by using the TruSeq DNA Methylation Kit (Illumina) according to the manufacturer’s instructions. Briefly, random hexamers were annealed to the bisulfite-converted single-stranded genomic DNA, complementary DNA were synthesized, DNA were tagged by terminal-tagging oligos whose 3′ end blocked, and then tagged DNA was purified. The library was amplified by using ten cycles of PCR amplification with indexing primers and was purified by using AMPure XP Beads (Beckman Coulter).

Paired-end WGBS libraries were sequenced on the Illumina NextSeq 500 or NovaSeq 5000 machine. The reads were de-multiplexed by using sample-specific index sequences. To increase the mapping efficiency, the first six low-quality base pairs of the sequence reads were trimmed along with adapter sequences by using Trim Galore! (The Babraham Institute) version 0.4.1 with the following parameters: --clip_R1 6 --clip_R2 6 --paired --retain_unpaired -r1 21 -r2 21. The trimmed reads were mapped to *in sillico* bisulfite-converted custom zebrafish genome reference (see below) by using Bismark [48] version 0.16.1 with the following parameters: -I 0 -X 2000 --un --ambiguous --bowtie2 -N 1 -L 28 --dovetail --score_min L,0,-0.6.

To improve the mapping efficiency, a custom zebrafish genome sequence for transgenic animal *Tg(sp7:EGFP)* was generated from all the next-generation sequencing data generated on this transgenic animal (H.J.L., T.W. and S.L.J., unpublished data) by using GATK’s variant call framework [49, 50] based on the zebrafish genome assembly GRCz10 (danRer10). This custom zebrafish genome sequence has a total of 3,660,642 single nucleotide variants of high confidence (0.26% of the total haploid genome length) from the reference GRCz10 assembly, disrupting 307,956 CpG sites (1.25% of total CpG sites). These disrupted CpG sites were excluded from further analyses. Small insertion or deletion variants were not included in the custom zebrafish genome. This custom zebrafish genome sequence was *in sillico* converted into a bisulfite-treated genome and was then used in Bismark mapping.

Unpaired or unmapped read 1s were then mapped as single read mode by using Bismark with the following parameters: --bowtie2 -N 1 -L 28 --score_min L,0,-0.6. Unpaired or unmapped read 2s were also mapped as single read mode by using Bismark with the following parameters: --pbat --bowtie2 -N 1 -L 28 --score_min L,0,-0.6.

The redundant reads from PCR amplification were then removed by using the following Bismark command: deduplicate_bismark -p --bam. Methylation levels of each C nucleotide were extracted from the de-duplicated reads by using Bismark with the following two commands: bismark_methylation_extractor --paired-end --no_overlap --comprehensive --report --gzip for paired-end mapped reads and bismark_methylation_extractor --single-end --comprehensive --report --no_header --gzip for reads mapped with single end mode. After merging paired-end and single-end report files, the Bismark command coverage2cytosine and a custom script were used to calculate total read counts and methylation read counts per each CpG. The methylation levels and read coverage of each CpG were visualized on the WashU Epigenome Browser [51] using a methylC track [52].

### Identification and analysis of DMRs

DMRs were identified by using the DSS pipeline [27, 28]. First, the mean methylation level of each CpG site was estimated with smoothing [53]. Then, dispersion at each CpG site was estimated, and a Wald test of each CpG site was performed to calculate statistical significance of methylation difference across different samples. Without replicates, DMRs were detected by calling DSS package’s callDMR function with a varying *p* value threshold and the following parameters: delta = 0, minlen = 200, minCG = 5, dis.merge = 50, pct.sig = 0.5. DMRs with each *p* value threshold were filtered by using the following criteria: average methylation differences of DMRs are bigger than 0.25. Final set of DMRs were chosen with *p* < 10^− 5^ and further filtered with at least 5 CpGs covered by at least 5 read counts. A different DMR calling algorithm, MethPipe [54], was used to identify DMRs independently to confirm our results. Default parameters were used for the following commands of MethPipe: hmr, methdiff, and dmr. DMRs with each *p* value threshold were filtered with similar criteria: (1) at least 3 CpGs in a DMR have at least 5 read counts; (2) average methylation differences of DMRs are bigger than 0.25.

Heatmaps and average line plots of DNA methylation and ATAC-seq signal levels of DMRs along with their neighboring regions were plotted using deepTools [55]. Methylation levels of each DMR in different samples were calculated by averaging smoothed methylation levels of CpGs inside the DMR.

### RNA-seq library generation, sequencing and mapping

RNA-seq libraries were generated by using TruSeq RNA Library Prep Kit v2 (Illumina), according to the manufacturer’s instructions. Briefly, mRNA was purified and fragmented from 500 ng of total extracted RNA. The first and second strands of cDNA were sequentially synthesized from the mRNA, the end of the cDNA was repaired, and indexing adapters were ligated. The library was amplified by using 15 cycles of PCR amplification and was purified by using the AMPure XP Beads (Beckrman Coulter).

Paired-end RNA-seq libraries were sequenced on the Illumina NextSeq 500 machine, with a total of ~ 400 million reads. The reads were de-multiplexed by using sample-specific index sequences. The sequence reads were mapped to the zebrafish transcriptome (Ensembl release 85) and the zebrafish genome assembly (GRCz10) by using STAR aligner [56] version 2.5.2a with the following parameters: --sjdbScore 1 --clip3pAdapterSeq AGATCGGAAGAGC --outWigStrand Unstranded --outFilterType BySJout --outFilterMultimapNmax 1. The number of reads mapped to each gene belonging to all genes from the Ensembl release 85 was summarized by using featureCounts [57] version 1.5.0 with the following parameters: -F GTF -t exon -g gene_id -O -s 0 --primary -p. The RNA expression levels as RPM (reads per million mapped reads) were visualized on the WashU Epigenome Browser [51] using bedGraph tracks generated by STAR.

### Identification and analysis of differentially expressed genes

Differential gene expression analysis was performed using DESeq2 [58] version 1.18.1. Genes with fold change > 2 and FDR < 0.05 were considered as significantly differentially expressed. Transcripts per million (TPM) was calculated for each gene from the number of reads mapped to each gene determined by featureCounts [57]. *Z*-scores were calculated by using rlog normalized read counts per gene among each comparison. Metascape [59] was used to analyze enriched GO terms for each category of differentially expressed genes. Distal enhancer elements of the differentially expressed genes were defined as ATAC-seq peaks located closest to the TSS, but also farther than 10 kb, by using BEDTools [60] version 2.27.1.

### ATAC-seq library generation, sequencing, and mapping

For ATAC-seq library generation, 3 uninjured fins and 10 blastema per replicate were collected from transgenic zebrafish *Tg(sp7:EGFP)* and *sp7*+ and *sp7*− cell populations were isolated by using FACS as described above. Approximately 70,000 cells were collected from FACS and immediately used for ATAC-seq library generation. ATAC-seq libraries were generated as previously described [35]. Briefly, 70,000 cells were washed with 50 μL of cold PBS buffer, lysed in 50 μL of cold lysis buffer (10 mM Tris, pH 7.4, 10 mM NaCl, 3 mM MgCl_2_ and 0.1% IGEPAL CA-630), and incubated with TDE1 enzyme (from Nextera DNA Sample Preparation Kit, Illumina) for 30 min at 37 °C for transposition. Transposed DNA fragments were immediately purified by using a MinElute PCR Purification Kit (Qiagen). ATAC-seq libraries were amplified by using 11 cycles of PCR amplification with an initial 5-min extension at 72 °C and purified by using AMPure XP Beads (Beckrman Coulter). The purified libraries were eluted with 20 μL of nuclease-free water.

Paired-end ATAC-seq libraries were sequenced on an Illumina NextSeq 500 machine, with a total of ~ 470 million reads. The reads were de-multiplexed by using sample-specific index sequences. Nextera adapter sequences were trimmed by using cutadapt [61] version 1.11. The trimmed reads were mapped to the custom zebrafish genome sequence (see above) by using bowtie2 [62] version 2.3.3.1 with the following parameters: --local -k 4 -X 2000 --mm. Secondary alignment, multiply mapped reads, and PCR duplicated reads were removed from the total aligned reads.

### Identification of ATAC peaks and DARs

The filtered aligned ATAC-seq reads were used to map to the transposon insertion sites, ATAC peaks were called per replicate from the insertion sites, and the irreproducible discovery rate (IDR) framework [63] version 2.0.4 was applied to identify highly reproducible ATAC peaks from two replicates.

First, reads mapped to mitochondrial DNA and unplaced scaffolds were removed from the aligned reads. Both ends of the paired-end reads were then treated as two Tn5 insertion sites. Tn5 insertion sites were adjusted to reflect the actual binding center of transposons as follows. All reads mapped to the + strand were offset by + 4 bp, and all reads mapped to the − strand were offset by − 5 bp.

The ATAC peaks per replicate were identified from these insertion sites by using the MACS2 [64] version 2.1.1 callpeak function with the following parameters: -g 1.34e9 --keep-dup all -B --SPMR --nomodel --extsize 73 --shift -37 -p 0.01 --call-summits. The IDR analysis was performed following ENCODE’s guidelines [65]. The ATAC peaks with IDR < 0.05 were chosen as highly reproducible accessible chromatin regions. The ATAC-seq signals were visualized on the WashU Epigenome Browser [51] as fold change over background using bedGraph tracks generated by using the MACS2 bdgcmp function with the following parameter: -m FE.

To identify DARs, DiffBind [36] version 2.6.6 was used on the union set of ATAC peaks with the following parameters: fragmentSize = 1, summits = 0. ATAC peaks with fold change > 2 and FDR < 0.01 were considered as significantly differentially accessible regions. The closest TSS per DAR was chosen by using BEDTools [60] version 2.27.1 and Ensembl gene annotation (release 85). Heatmaps and average line plots of DNA methylation and ATAC-seq signal levels of DARs along with their neighboring regions were plotted by using deepTools [55]. Methylation levels of each DAR in different samples were calculated by averaging smoothed methylation levels of CpGs inside the DAR.

### In vivo activity validation of regeneration enhancers

The original ZED vector [41] was modified by replacing the transgenesis internal control cassette (*cardiac_actin_promoter:dsRed*) with the strong constitutive marker (*EF1α:mCherry*). The vector was further modified by replacing the *gata2* minimal promoter with the 2-kb promoter sequence of the zebrafish *lepb* gene [33]. The modified ZED vector was named ZEDtw plasmid, and the sequence was verified by Sanger sequencing.

Candidate enhancer elements and negative control sequences were PCR amplified from zebrafish genomic DNA using primers listed in Additional file 1: Table S4. The amplified elements were then cloned into ZEDtw vector by the gateway in vitro recombination system as described previously [41]. Purified plasmids were then injected into wildtype TU zebrafish embryo at one-cell stage. All mCherry+ F_0_ embryos were raised up to adult. For some enhancer elements, F_1_ stable lines were also established by outcrossing F_0_ with wildtype TU zebrafish. Once F_0_ or F_1_ zebrafish were raised to adult, caudal fins were amputated. The EGFP and mCherry expressions were monitored and photographed on the regenerating fin every day up to 4 dpa.

### Construction of gene regulatory networks

Motif enrichment analysis on DARs was performed using HOMER [66] version 4.8. HOMER scanned the sequences of DARs for known motifs, and calculated enrichment score *p* values using a binomial test. HOMER also discovered de novo motifs with their best matches to a known motif in DARs.

Footprint analysis was performed by CENTIPEDE [67]. First, instances of a given motif were identified across the entire zebrafish genome using FIMO [68] version 4.11.2 with the following parameters: --max-stored-scores 10000000 --text --thresh 1e-5. Then Tn5 insertion events from ATAC-seq in 200 bp windows around these motif sites were counted. These count matrices were then used as input for CENTIPEDE along with the conservation scores (phastCons scores from 8-way vertebrate genome alignment, lifted over from Zv9 to GRCz10) at corresponding positions to predict the likelihood that each motif instance is bound by a TF. The motif instances with posterior probability greater than 0.95 were used as TF-bound sites.

To build putative regulatory network, TFs were linked to their downstream target genes, with a similar method as the one described previously [20]. First, TFs whose motifs were enriched in DARs that gained accessibility during regeneration either in *sp7*+ or in *sp7*− cells were chosen by HOMER as described above. Then, the expression levels of the zebrafish ortholog genes corresponding to those TFs were examined. Among them, TFs whose expression levels were higher in regenerates at 1, 2, or 4 dpa than in uninjured fins were further selected as putative upstream factors. Their new bound sites in fin regenerates at 4 dpa were identified by intersecting TF-bound sites predicted by CENTIPEDE and DARs that gained accessibility during regeneration. Finally, TFs with those new bound sites in fin regenerates at 4 dpa were chosen to build putative regulatory network. The closest TSS from each new bound site was identified by BEDTools [60], and it was classified as a putative downstream target gene of a given TF if that closest gene was upregulated during regeneration. Putative TF-TF regulations and TF auto-regulation were also identified in case that a putative target gene was one of the chosen TF. The enriched GO terms from Metascape analysis were used to annotate the putative downstream target genes.

### Generation of mutant zebrafish with CRISPR/Cas9 genome editing

Mutant fish strains were generated by a customized CRISPR/Cas9-based targeted genome editing system (H.S.J., T.W. and S.L.J., in preparation). All gRNAs targeting the *fosl1a* gene were scored by the formula (Score = 60 × [proportion GC content] + 10 × [proportion transcripts targeted] − 30 × [relative position in gene] + 2[if position 20 is G] − 3[if position 20 is A]) and ranked by this score as well as the number of predicted off target effects. The top four gRNAs were selected, and their sequences were cloned downstream of each of the four U6 promoters on the pT2-U6chr21-gRNAscaffold vector via Gibson assembly (NEB, E2611S). The modified vector was transformed into TOP10 competent cells (Thermo Fisher, C404010) and extracted by using a Hi-Speed Mini Plasmid Kit (IBI, IB47102). Wildtype AB zebrafish embryos were injected with a 1 nL mixture containing the modified vector (90 ng/μL) and the Tol2 transposase mRNA (30 ng/μL) at one-cell stage. The GFP-positive F_1_ from the crossing with *Tg(hsp70:zCas9; mylz:CFP)* were collected at 1 day post fertilization and heat shocked at 37 °C for half an hour to induce genome editing. Heterozygous F_2_ zebrafish founders were generated by outcrossing mosaic F_1_ mutants with wildtypes and individual genotyping on the gRNA target site. Heterozygous F_2_ mutant founders carrying the same *fosl1a* mutant allele were inter-crossed to generate homozygous mutant zebrafish for regenerative morphometric measurements.

### Measurement of fin regenerate lengths

For each batch of mutant and wildtype littermates from one pair of heterozygous parents (*fosl1a*^+/−^), the caudal fins were amputated halfway along the proximal-distal axis. Their regeneration processes were photographed daily up to 5 dpa. The lengths of the new regenerates were measured with ImageJ (NIH), from the cutting plane to the front end. The fourth fin ray counting from the ventral side was used for consistency. The measurements were taken from distinct samples, and the exact sample size for each group is indicated in the figures.

### Quantitative reverse transcription (qRT)-PCR

The primers for qRT-PCR were designed targeting regions spanning multiple exons per each gene (Additional file 2: Table S4). First-strand cDNA was synthesized from 1 μg of total RNA by using ProtoScript II Reverse Transcriptase (NEB, M0368) according to the manufacturer’s instructions. A standard qRT-PCR on the cDNA corresponding to 25 ng of total RNA was performed using PerfeCTa SYBR Green SuperMix (Quantabio, 95054) and Bio-Rad CFX96 machine. The gene expression levels were normalized using *actb2* gene expression level as an internal control.

### Statistics

All the statistical tests were performed by using R program version 3.4.3. A two-sided Mann–Whitney *U* test was used to test statistical differences of fin regenerate lengths between mutant and wildtype zebrafish, by using the wilcox.test function. The two-sided Wilcoxon signed-rank test was used to test statistical differences of gene expression changes in regenerates between mutant and wildtype zebrafish, by using the wilcox.test function with the parameter: paired = T. Sample sizes and *p* values are indicated in the figures or figure legends.

## Supplementary information

Additional file 1: Figure S1. DNA methylation is stably maintained during zebrafish fin regeneration. Figure S2. DNA methylome maps of *sp7*+ and *sp7*− cells during fin regeneration. Figure S3. Transcriptome maps of *sp7*+ and *sp7*− cells during fin regeneration. Figure S4. Chromatin accessibility maps identify regeneration-specific enhancers. Figure S5. Comparison of regeneration-specific DARs and Phylo(−)DMRs. Figure S6. Gene regulatory networks identify upstream factors for fin regeneration.

Additional file 2: Table S1. Overview of sequencing and mapping in this study. Table S4. Primer sequences used in this study.

Additional file 3: Table S2. Differentially expressed genes during fin regeneration. **a** Upregulated genes in *sp7*+ cells during fin regeneration. **b** Downregulated genes in *sp7*+ cells during fin regeneration. **c** Upregulated genes in *sp7*− cells during fin regeneration. **d** Downregulated genes in *sp7*− cells during fin regeneration.

Additional file 4: Table S3. Differentially accessible regions during fin regeneration. **a** DARs with increasing signals in *sp7*+ cells during fin regeneration. **b** DARs with decreasing signals in *sp7*+ cells during fin regeneration. **c** DARs with increasing signals in *sp7*− cells during fin regeneration. **d** DARs with decreasing signals in *sp7*− cells during fin regeneration.

Additional file 5. Review history.

## Data Availability

The datasets generated and analyzed during the current study are available in the NCBI’s Gene Expression Omnibus (GEO) repository (ATAC-seq: GSE126700 [69], RNA-seq: GSE126701 [70], and WGBS: GSE126702 [71]). All sequencing data processed in the current study have been visualized in the WashU Epigenome Browser and is publicly available through the following url: https://epigenome.wustl.edu/FinRegen/

## Authors’ information

Twitter handles: Hyung Joo Lee @hyungjoo_lee

Yiran Hou @ h_yiran

Yujie Chen @YujieChen19 

Ting Wang @twang5
