# Supplementary material for: Regenerating zebrafish fin epigenome is characterized by stable lineage-specific DNA methylation and dynamic chromatin accessibility
Source: Genome Biol. 2020 Feb 27;21:52. doi: 10.1186/s13059-020-1948-0 (PMC7047409; doi:10.1186/s13059-020-1948-0)
Supplement: Supplementary file 1 — Figure S1. DNA methylation is stably maintained during zebrafish fin regeneration. Figure S2. DNA methylome maps of sp7+ and sp7− cells during fin regeneration. Figure S3. Transcriptome maps of sp7+ and sp7− cells during fin regeneration. Figure S4. Chromatin accessibility maps identify regeneration-specific enhancers. Figure S5. Comparison of regeneration-specific DARs and Phylo(−)DMRs. Figure S6. Gene regulatory networks identify upstream factors for fin regeneration. [file 13059_2020_1948_MOESM1_ESM.pdf]

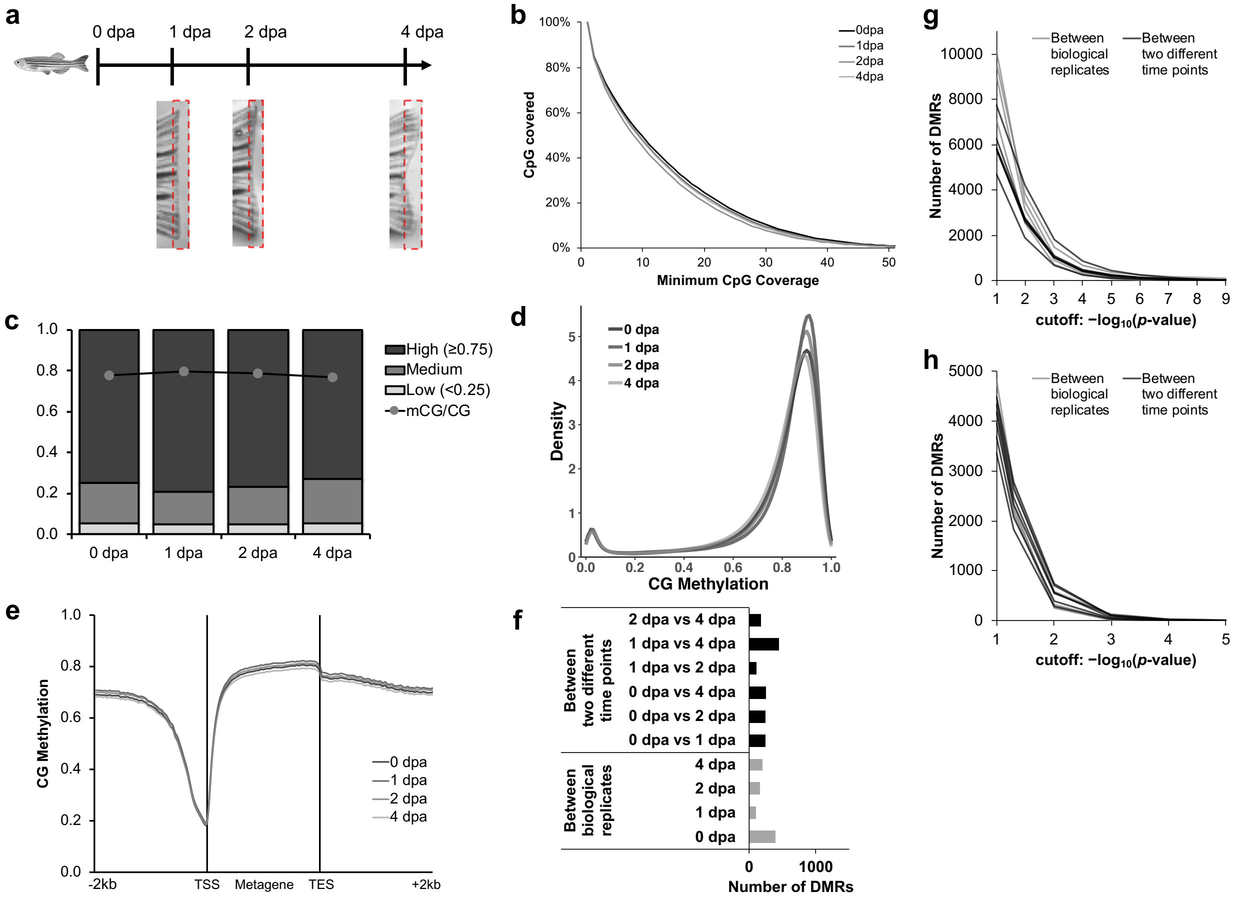

Figure S1 DNA methylation is stably maintained during zebrafish fin regeneration. **a** Timeline for collecting uninjured (0 dpa) and regenerating fins (1, 2, and 4 dpa). Representative zebrafish fin regenerates from the same animal at three different time points were shown. The regenerates in the red dotted boxes were collected to extract genomic DNA. dpa, days post amputation. **b** Percentage of CpGs covered at different minimum coverage. More than 60% of CpGs were covered at least 5× coverage depth. **c** Global CpG methylation levels (mCG/CG, black line) and fraction of total CpGs with low (<25%), medium (≥25% and <75%), and high (≥75%) methylation levels at different time points during zebrafish fin regeneration. **d** Distribution of genome-wide CpG methylation levels at each time point. **e** Average CpG methylation levels over the protein coding genes and neighboring 2 kb regions. TSS, transcription start site; TES, transcription end site. **f** Number of DMRs identified between two different time points (black bars) or between two biological replicates (grey bars). **g** Number of DMRs identified at different p-value cutoffs used in DSS. DMRs were called between biological replicates (grey lines), and between two different time points (black lines). **h** Number of DMRs identified at different p-value cutoffs used in MethyPipe. DMRs were called between biological replicates (grey lines), and between two different time points (black lines).

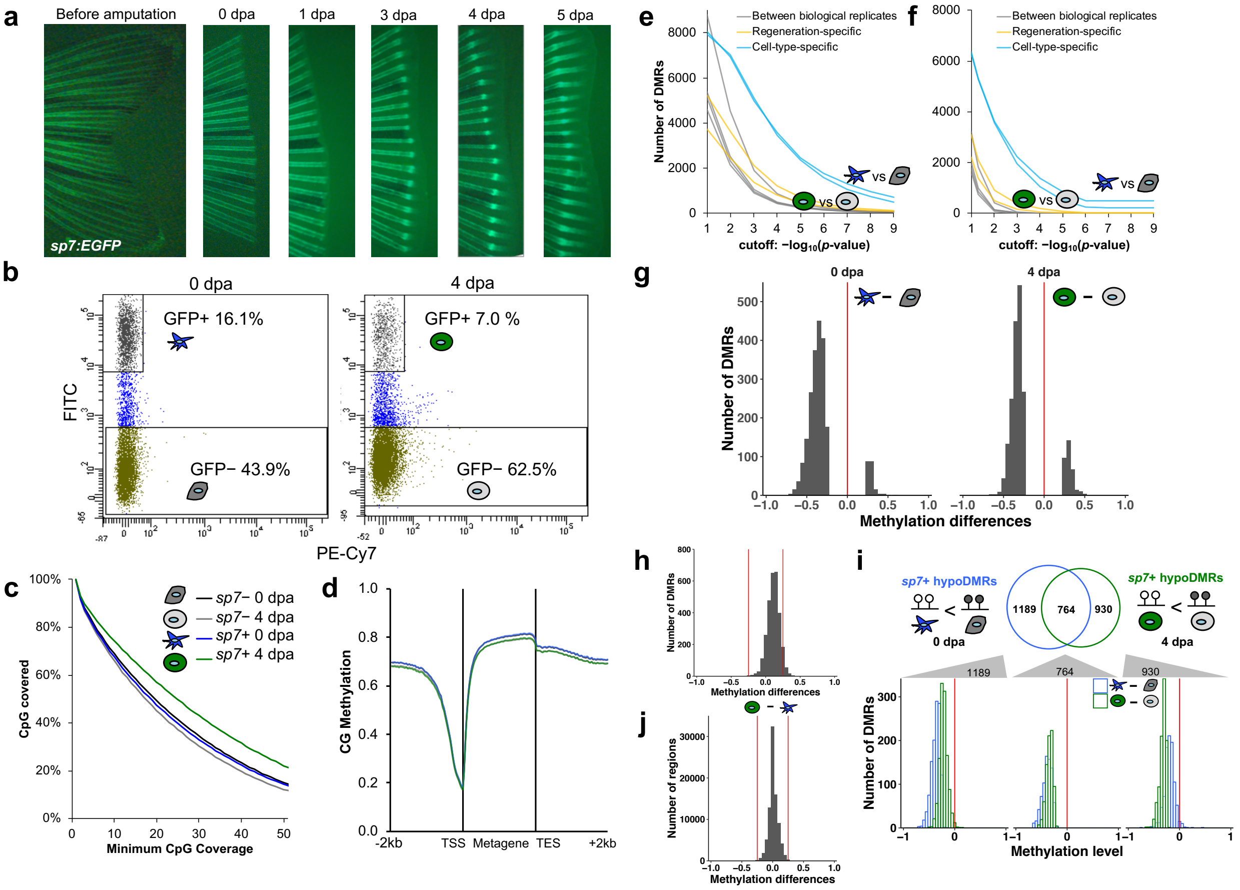

Figure S2 DNA methylome maps of *sp7*+ and *sp7*– cells during fin regeneration. **a** Time course expression of *sp7:EGFP* in regenerating fins. Amputated fin of the same *sp7:EGFP* transgenic fish from 0 day to 5 days post amputation (dpa). *sp7:EGFP* expression is observed in osteoblasts located on the surface of the bony rays. EGFP is strongly expressed at 3 dpa in regenerates. **b** Separation and isolation of *sp7*+ and *sp7*– cells from *Tg(sp7:EGFP)* zebrafish. Representative FACS plots of dissociated cells from 0 dpa uninjured fins (left) and 4 dpa blastema (right). X-axis is arbitrary PE-Cy7 fluorescence values for violet color. Y-axis is arbitrary FITC fluorescence values for EGFP. Each dot represents a single cell. A rectangular gate was made to select either GFP+ (top gate) or GFP– cells (bottom gate). **c** Percentage of CpGs covered at different minimum coverage. Around 80% of CpGs were covered at least 5× coverage depth. **d** Average CpG methylation levels over the protein coding genes and neighboring 2 kb regions. TSS, transcription start site; TES, transcription end site. **e** Number of DMRs identified at different *P*-value cutoffs used in DSS. DMRs were called between two biological replicates (grey lines), between 0 dpa and 4 dpa in the same cell type (regeneration-specific, yellow lines), and between *sp7*+ and *sp7*– cells at the same time point (cell-type-specific, blue lines). **f** Number of DMRs identified at different p-value cutoffs used in MethPipe. DMRs were called between two biological replicates (grey lines), between 0 dpa and 4 dpa in the same cell type (regeneration-specific, yellow lines), and between *sp7*+ and *sp7*– cells at the same time point (cell-type-specific, blue lines). **g** Histogram of DMRs with DNA methylation differences. Methylation differences were calculated as DNA methylation level of *sp7*– cells subtracted from DNA methylation level of *sp7*+ cells. Most DMRs have negative values for methylation differences, suggesting that most of them are hypoDMRs in *sp7*+ cells. **h** Histogram of *sp7*+ hypoDMRs with DNA methylation differences during regeneration. Methylation differences were calculated as DNA methylation level of *sp7*+ cells at 0 dpa subtracted from DNA methylation level of *sp7*+ cells at 4 dpa. **i** Venn diagram of two *sp7*+ cell-specific hypoDMRs (blue and green circles for 0 dpa and 4 dpa, respectively) intersecting with each other (top) and histograms of their DNA methylation differences between *sp7*+ and *sp7*– cells (bottom). All three categories of hypoDMRs displayed negative values of DNA methylation differences, indicating that those regions are *sp7*+ cell-specific hypoDMRs. **j** Histogram of the lowly methylated regions in *sp7*+ cells with DNA methylation differences during regeneration. Methylation differences were calculated as DNA methylation level of *sp7*+ cells at 0 dpa subtracted from DNA methylation level of *sp7*+ cells at 4 dpa.

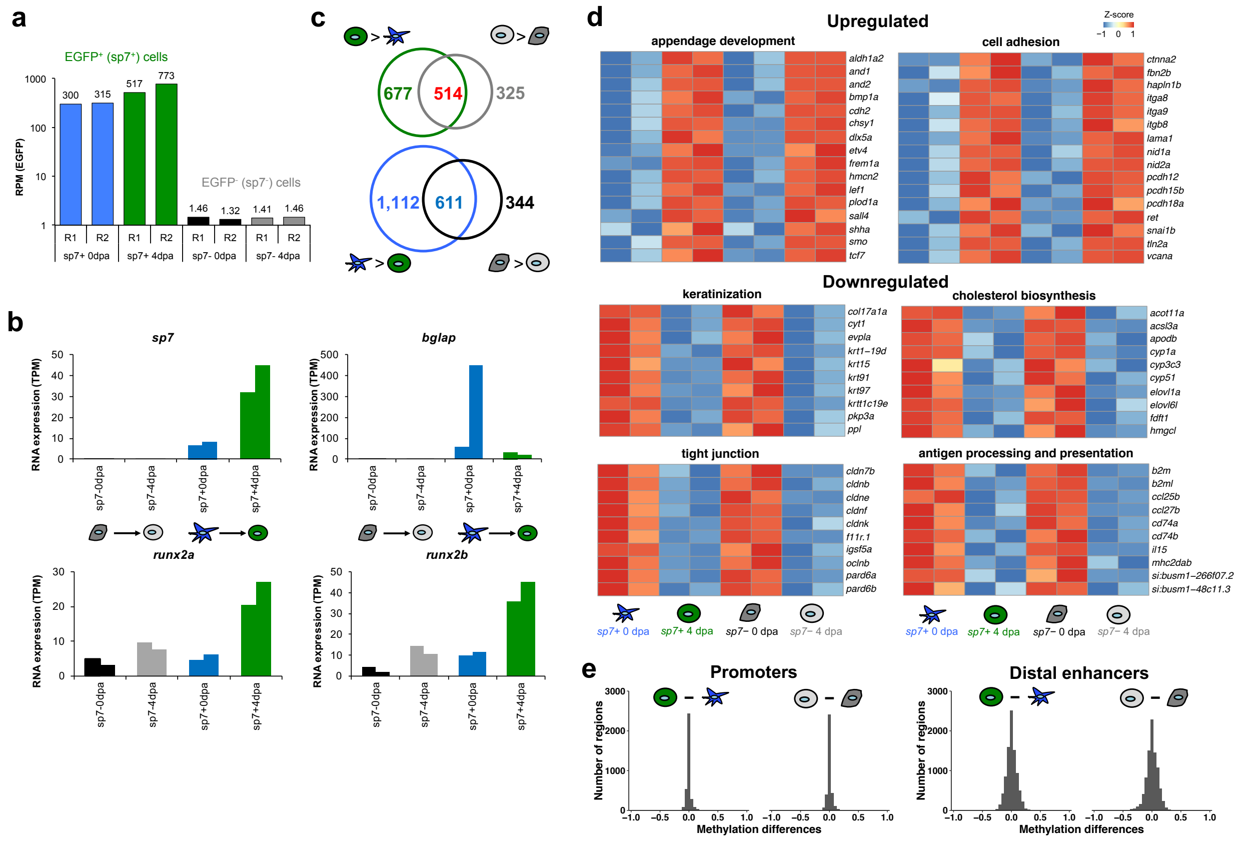

Figure S3 Transcriptome maps of *sp7*+ and *sp7*– cells during fin regeneration. **a** Transcript abundance of EGFP in each sample. R1, replicate 1; R2, replicate 2. **b** Expression levels of osteoblast lineage-specific genes in each sample. **c** Venn diagram showing the numbers of significantly differentially expressed genes during fin regeneration. **d** Expression levels of example genes upregulated or downregulated during regeneration in both *sp7*+ and *sp7*– cells. **e** Histogram of promoters (left) and distal enhancers (right) whose genes are differentially expressed during regeneration with their DNA methylation differences. Methylation differences were calculated as DNA methylation level at 0 dpa subtracted from DNA methylation level at 4 dpa. Majority of the regions have little differences in DNA methylation levels, suggesting that the DNA methylation levels were stably maintained in these regulatory regions during regeneration.

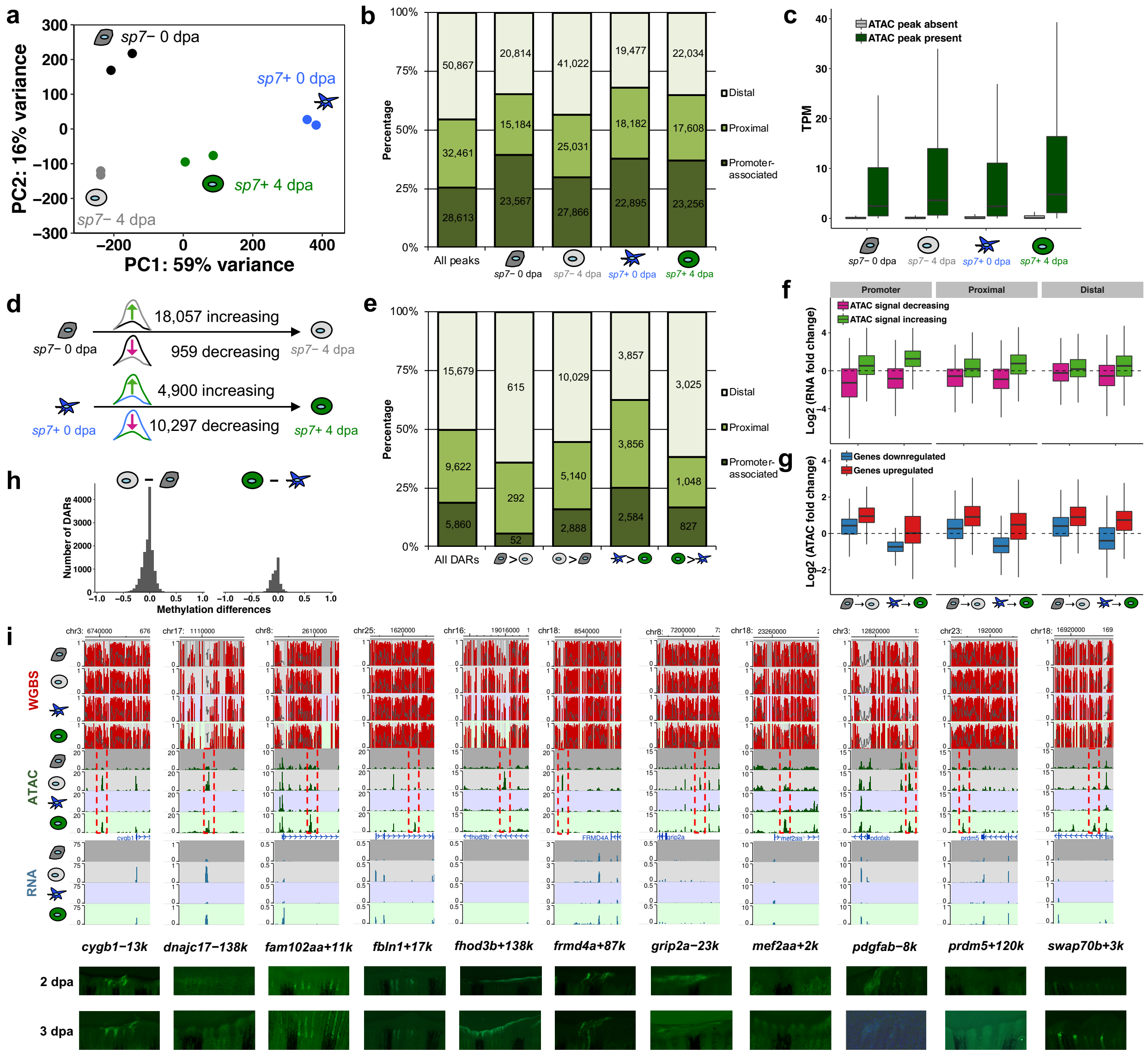

Figure S4 Chromatin accessibility maps identify regeneration-specific enhancers. **a** Principle component analysis on the chromatin accessibility of *sp7*+ and *sp7*– cells at 0 dpa uninjured fin and 4 dpa blastema. **b** Percentage of ATAC peaks that are promoter-associated (located ≤1 kb from TSS), proximal (located ≤10 kb from TSS), or distal. Numbers indicate the number of ATAC peaks belonging to each category. **c** Promoters that are accessible (dark green boxes) have an increased abundance of RNA transcripts, while promoters without ATAC peaks (grey boxes) have little or no RNA expression. TPM, transcripts per million. **d** Number of DARs identified in *sp7*+ and *sp7*– during regeneration. **e** Percentage of DARs that are promoter-associated (located ≤1 kb from TSS), proximal (located ≤10 kb from TSS), or distal. Numbers indicate the number of DARs belonging to each category. **f** Expression changes are shown as fold change for the genes which DARs are located nearby. **g** ATAC-seq signal changes are shown as fold change for the ATAC peaks whose closest genes have differential expression during regeneration. **h** Histogram of DARs that gained chromatin accessibility with their DNA methylation differences. Methylation differences were calculated as DNA methylation level at 0 dpa subtracted from DNA methylation level at 4 dpa. Majority of the DARs have very small methylation differences, suggesting that the DNA methylation levels were stably maintained in DARs during regeneration. **i** Epigenome browser views of regeneration enhancers (top) and transgenic zebrafish showing enhancer activities in the regenerating fin (bottom). Red dashed boxes indicate DARs that gained accessibility during regeneration.

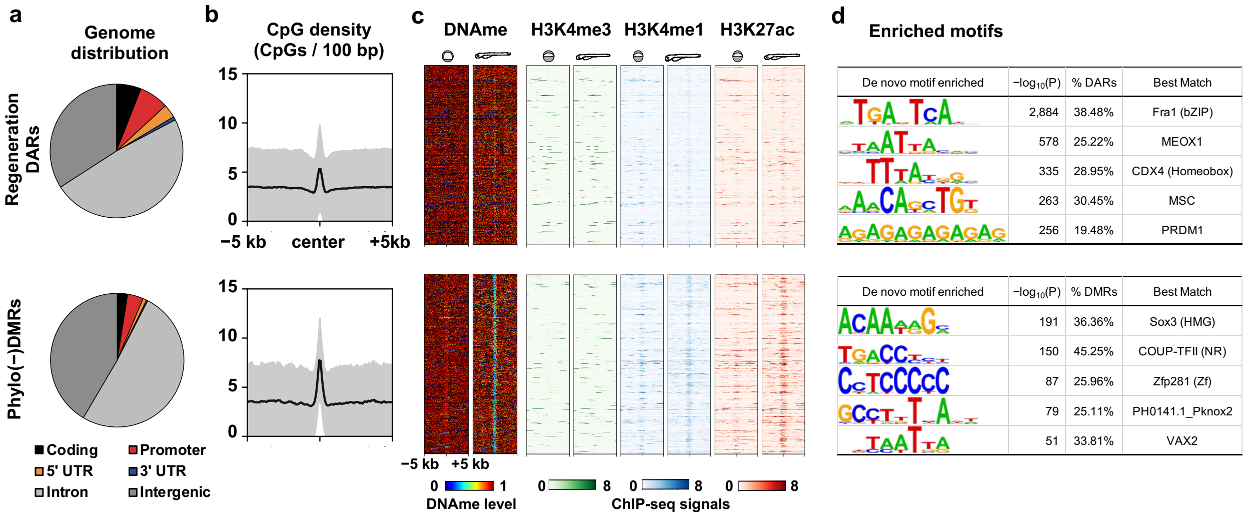

Figure S5 Comparison between the regeneration-specific DARs and Phylo(−)DMRs. **a** Pie charts of the genomic location of the two sets of regulatory elements. **b** Mean CpG densities of the 10-kb neighboring regions around the regulatory elements. Shaded areas represent standard deviation. **c** heat maps of DNA methylation levels and normalized ChIP-seq signals of H3K4me3, H3K4me1, and H3K27ac at the 10-kb neighboring regions around the regulatory elements during zebrafish development. **d** Enriched de novo motifs in the regulatory elements detected by HOMER.

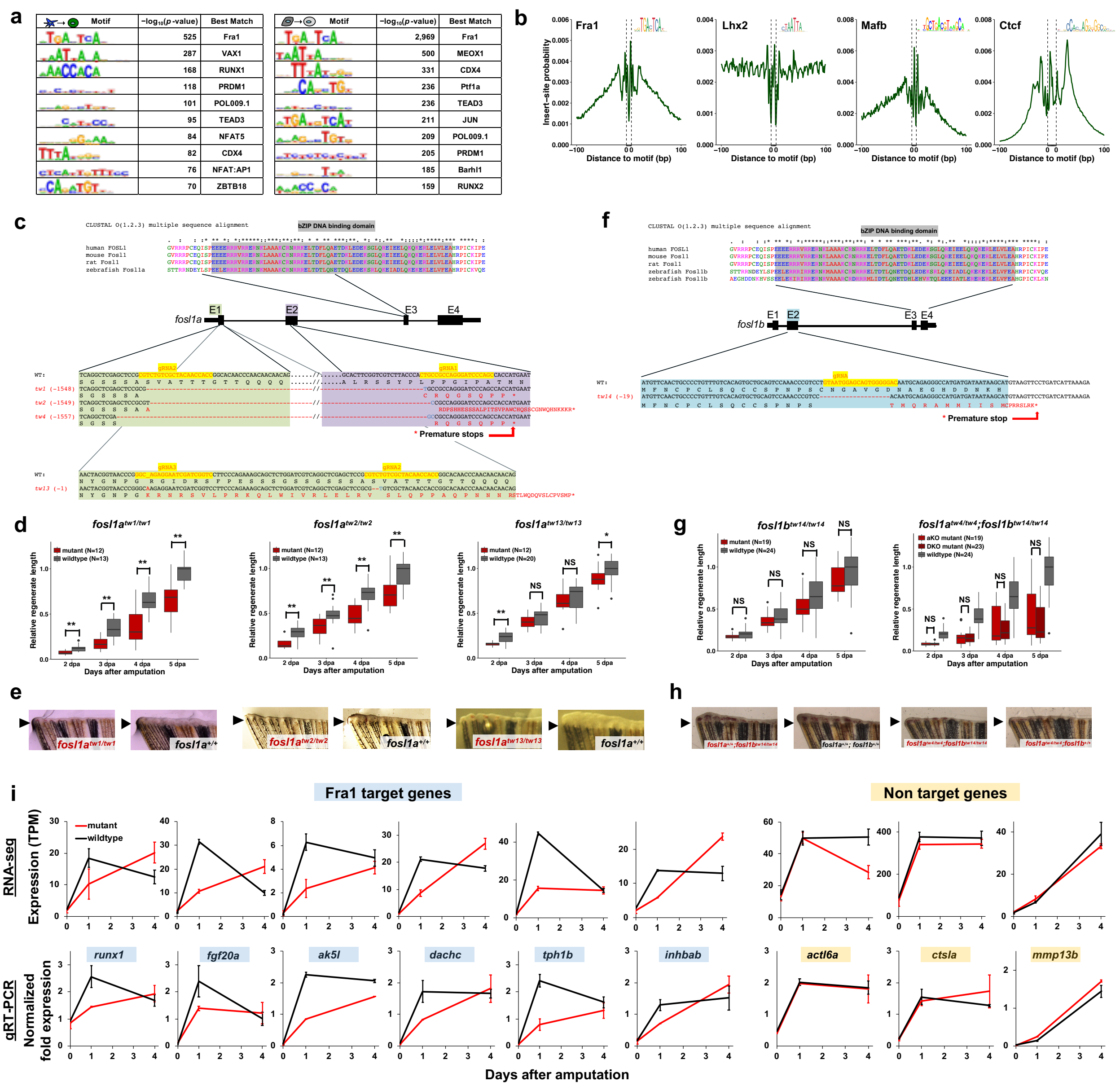

Figure S6 Gene regulatory networks identify upstream factors for fin regeneration. **a** Top 10 HOMER de novo motifs found in DARs that gained accessibility during regeneration in *sp7*+ (left) and *sp7*– cells (right) and their best matches to a known motif. **b** ATAC-seq footprints of the TFs whose motifs were enriched in DARs. **c** CRISPR-Cas9 genome editing introduced mutations that disrupt DNA binding domain of Fra1 protein. The nucleotide sequences (bottom) of the wildtype *fosl1a* gene and 4 different mutant alleles (*tw1*, *tw2*, *tw4*, and *tw13*) generated by CRISPR-Cas9 genome editing. The amino acid sequences translated from wildtype or mutant allele were shown below each nucleotide sequence. The bZIP DNA binding domain (grey box) which is evolutionarily highly conserved as shown on top is located in exon 3 and exon 4. The mutations were made in the exon 2 (green box) and exon 3 (purple box), disrupting DNA binding domain by introducing premature stop codons (*) upstream of it. **d** Boxplots showing fin regenerate lengths as a function of the time after amputation. Mutant zebrafish (red) showed delayed regeneration after fin amputation compared to their wildtype littermates (grey). Left, *fosl1a*$^{tw1/tw1}$; center, *fosl1a*$^{tw2/tw2}$; right, *fosl1a*$^{tw13/tw13}$. $*P < 0.1$; $**P < 0.01$; NS, not significant; Mann–Whitney *U* test. **e** Representative pictures of fin regenerate from the *fosl1a* mutant zebrafish with their wildtype (*fosl1a*$^{+/+}$) littermates at 2 dpa. Arrowhead, amputation plane. **f** CRISPR-Cas9 genome editing introduced mutations that disrupt DNA binding domain of Fra1 protein. The nucleotide sequences (bottom) of the wildtype *fosl1b* gene and mutant alleles (*tw14*) generated by CRISPR-Cas9 genome editing. The amino acid sequences translated from wildtype or mutant allele were shown below each

nucleotide sequence. The bZIP DNA binding domain (grey box) which is evolutionarily highly conserved as shown on top is located in exon 3 and exon 4. The mutations were made in the exon 2 (blue box), disrupting DNA binding domain by introducing premature stop codons (*) upstream of it. **g** Boxplots showing fin regenerate lengths as a function of the time after amputation. Mutant zebrafish (red) showed delayed regeneration after fin amputation compared to their wildtype littermates (grey). Left, *fosl1b*$^{tw14/tw14}$; right, *fosl1a*$^{tw4/tw4}$;*fosl1b*$^{+/+}$ (aKO) and *fosl1a*$^{tw4/tw4}$;*fosl1b*$^{tw14/tw14}$ (DKO). NS, not significant; Mann–Whitney *U* test. **h** Representative pictures of fin regenerate from the *fosl1b* mutant zebrafish with their wildtype (*fosl1b*$^{+/+}$) littermates in either fosl1a mutant or wildtype background at 2 dpa. Arrowhead, amputation plane. **i** Expression levels of several example Fra1 target genes (*runx1*, *fgf20a*, *ak5l*, *dachc*, *tph1b*, and *inhbab*) and non-target genes (*actl6a*, *ctsla*, and *mmp13b*). Top, expression levels in TPM from RNA-seq. Bottom, Expression levels of these genes were validated by qRT-PCR.
