# Supplementary material for: Regenerating zebrafish fin epigenome is characterized by stable lineage-specific DNA methylation and dynamic chromatin accessibility
Source: Genome Biol. 2020 Feb 27;21:52. doi: 10.1186/s13059-020-1948-0 (PMC7047409; doi:10.1186/s13059-020-1948-0)
Supplement: Supplementary file 2 — Table S1. Overview of sequencing and mapping in this study. Table S4. Primer sequences used in this study. [file 13059_2020_1948_MOESM2_ESM.pdf]

a

Sample	Replicate	Total reads	Mapped reads	Uniquely mapped reads	Total usable deduplicated reads	Conversion rate	CpG coverage	CpG coverage when merged 2 replicates
0 dpa	rep1	97,658,873	79,705,299	61,725,695	52,904,004	99.4%	5.4 ×	11.8 ×
	rep2	116,021,637	94,788,726	73,313,528	60,642,787	99.4%	6.4 ×	
1 dpa	rep1	92,058,825	76,114,617	58,906,303	51,640,789	99.4%	5.3 ×	11.4 ×
	rep2	113,204,853	91,046,108	70,075,098	58,299,260	99.4%	6.1 ×	
2 dpa	rep1	99,980,528	82,032,356	63,422,729	54,284,784	99.4%	5.6 ×	10.5 ×
	rep2	89,146,117	71,400,051	54,668,950	45,628,590	99.4%	4.9 ×	
4 dpa	rep1	101,604,298	83,685,983	64,884,467	55,677,089	99.4%	5.8 ×	11.2 ×
	rep2	101,359,139	81,288,749	62,543,423	50,097,492	99.4%	5.4 ×	
sp7 − 0 dpa	rep1	339,204,355	263,254,635	208,856,633	107,842,698	99.2%	16.2 ×	24.5 ×
	rep2	183,079,036	144,419,774	111,456,372	79,061,213	99.1%	8.3 ×	
sp7 − 4 dpa	rep1	321,079,453	248,605,943	193,871,449	90,207,873	99.0%	13.7 ×	22.2 ×
	rep2	186,176,940	147,505,796	114,235,979	80,567,036	98.9%	8.5 ×	
sp7 + 0 dpa	rep1	310,404,583	239,316,840	186,915,989	97,084,337	99.0%	14.6 ×	23.8 ×
	rep2	193,099,532	153,731,695	119,345,224	86,914,170	99.0%	9.2 ×	
sp7 + 4 dpa	rep1	425,545,301	327,149,583	258,216,194	142,611,326	99.0%	19.7 ×	29.9 ×
	rep2	216,557,384	172,815,147	134,644,393	98,949,557	99.0%	10.3 ×	

b

Sample	Replicate	Total reads	Mapped reads	Uniquely mapped reads
sp7 − 0 dpa	rep1	42,397,152	40,860,711	38,709,047
	rep2	51,447,151	49,646,142	46,650,676
sp7 − 4 dpa	rep1	56,263,069	54,220,674	50,111,039
	rep2	51,709,789	49,666,231	46,321,124
sp7 + 0 dpa	rep1	34,910,498	33,011,263	29,830,095
	rep2	34,659,048	32,667,395	29,230,285
sp7 + 4 dpa	rep1	56,330,334	54,353,845	49,679,693
	rep2	42,199,811	40,692,739	37,499,675
fosl1a WT 0 dpa	rep1	27,336,562	21,922,819	20,945,083
	rep2	28,693,638	25,782,283	25,020,842
fosl1a WT 1 dpa	rep1	38,003,475	33,259,462	32,286,064
	rep2	34,312,611	28,591,367	27,768,866
fosl1a WT 4 dpa	rep1	25,979,519	22,304,260	21,459,157
	rep2	29,289,437	25,747,832	24,772,616
fosl1a KO 0 dpa	rep1	14,486,097	11,394,027	10,966,548
	rep2	30,284,650	26,466,562	25,600,780
fosl1a KO 1 dpa	rep1	21,277,366	16,666,701	16,084,880
	rep2	24,836,141	21,148,430	20,335,083
fosl1a KO 4 dpa	rep1	32,587,700	30,137,052	28,905,772
	rep2	28,891,618	25,895,332	25,067,084

c

Sample	Replicate	Total reads	Mapped reads	Uniquely mapped reads	Deduplicated reads	Usable non-mtDNA deduplicated reads	NRF	PBC1
sp7 − 0 dpa	rep1	48,777,880	46,752,872	30,360,005	20,256,036	18,992,120	0.84	0.83
	rep2	52,228,548	50,206,758	30,530,025	25,436,495	24,599,307	0.90	0.90
sp7 − 4 dpa	rep1	65,295,695	62,852,594	41,481,708	35,512,644	34,610,284	0.92	0.92
	rep2	49,949,977	48,153,800	32,503,895	26,801,632	25,828,844	0.92	0.92
sp7 + 0 dpa	rep1	54,540,740	52,520,180	36,832,014	29,025,584	28,125,390	0.84	0.85
	rep2	45,460,131	43,822,103	30,220,217	24,286,404	23,372,268	0.87	0.88
sp7 + 4 dpa	rep1	54,873,697	52,880,959	33,498,301	27,758,854	26,892,808	0.90	0.90
	rep2	62,733,310	59,781,317	37,735,807	31,516,024	30,613,833	0.90	0.91

Table S1 Overview of sequencing and mapping in this study. **a** Overview of WGBS sequencing and mapping. **b** Overview of RNA-seq sequencing and mapping. **c** Overview of ATAC-seq sequencing and mapping. NRF, non-redundant fraction; PBC, PCR bottlenecking coefficient. NRF is defined as number of PCR deduplicated reads divided by the total number of aligned reads. PBC1 is defined as number of genomic coordinates mapped with only one unique read divided by total number of genomic coordinates mapped with all reads.

a

Primer name	Primer sequence	Usage
ins-EF1a-F2	GCACATTGGCTGCGAGCAGGGTGGATCATC	*EF1α:mCherry* fragment amplification
ins_mCh_R2	TCAGGAACAGGTGGccagtgtgatggatatctgcag	
vec_mCh_F2	GCGGCACGGAgtggagcagtacgagcg	*GAB* insulator fragment amplification from ZED
PacIZEDdown_GAB_R	CGCGCGTACGCCATGGTTAATCGCAATTAACCCTCACTAAAGG	
Ins_lepb2kb_F	CTCGAGGTCGACGGTATTCTTGAACAAGTGACTTTCGTTGCAAC	*lepb* promoter amplification from genomic DNA
Ins_lepb2kb_R	GACCGGTGGATCATTTCTGCAAAAGACCAAATGAAATTATATTTTTTC	
Vector_lepb2kb_F	CATTTGGTCTTTTGCAGAAATGATCCACCGGTCGCCACCATG	*EGFP* fragment amplification from ZED
Vec_GABIns-lepb2kb_R	CGGGGGATCCGAGCTCGGTACCAGCTTTTGTTCCCTTTAGTGAG	
Vec_CmR-lepb2kb_F	CTGGTGAAACTCACCCAGGGATTGGCTGAGACGAAAAACATATTCTCAATAAACC	*attR-CmR* fragment amplification from ZED
Vector_lepb2kb_R	ACGAAAGTCACTTGTTCAAGAATACCGTCGACCTCGAGATAAC	

b

Enhancer name	Genomic coordinate (GRCz10)	Length	Forward PCR primer sequence	Reverse PCR primer sequence
bmp2a+43k	chr17:4030142-4030727	586	CCAGTTAACCCACATGCTCA	AATATTCCCCGTTCCAGCCC
cdc42ep3+2k	chr13:42000312-42001230	919	GCTGTAATTGCTGCCAGTAAGT	CCACAGTGGCATCTCTTACC
chst3b+5kb	chr12:48989963-48990631	669	GCATTTACTGGAGCTCTGCC	AACCTCAACCTCCCCTGATG
col11a1a−6k	chr24:28819596-28820156	561	TTGACCTGAAGTGCAATGGT	GTTGTTCTTTCAGCAGGGCC
col11a2−7k	chr19:7312885-7313512	628	ATGACAGACGTTTCCCACCT	GCCGGTCTCCAAAATCACTG
cygb1−13kb	chr3:6740622-6741421	800	AAGCAACCAGAGTCAGACCA	CACATGAGATGAAGCGCTGG
dnajc17−138kb	chr17:1110676-1111231	556	TGACTGACCTCTACATACACCTG	GTCTGTGATTGGACGAGCTC
fam102aa+11k	chr8:2608089-2608632	544	TCGGCTGCTGATTACAATAAACT	ACACACCGCACAGAACCT
fbln1+17k	chr25:1624921-1625612	692	TCAGCATTATCAACACAACCGA	AGTCATCCAGAGTTGTTTATGTTGA
fhod3b+138k	chr16:19015751-19016085	335	GACTGCACAACTCATCTGTCC	CAGGTTCTGTGTTTTCATCATGT
frmd4a+87k	chr18:8534404-8534871	468	ATTAAAACAGGTGAGAAAAGAGCA	TCCCTTGCTCTGTTAAACATCA
grip2a−23k	chr8:7211666-7212152	487	CACACACACTTTTGCAGGGA	TGGCAACCCTTATCAGCTCT
igflr1−2k	chr15:37533052-37533452	401	TCAACTTGTGTGGATTCAGGA	ACCTTGTCTGGCTTAATTCTTGT
lef1−2k	chr1:49168747-49169031	285	CTGTGTCTCCTTTACCCGGA	CGTAGCAGAAAACAAGAAGGGG
mef2aa+2kb	chr18:23261921-23262551	631	ACACAGCAAACACAGCAAGC	AGCGTGAGTAGACAAGGGTG
mitd1−6k	chr9:7314150-7314957	808	AAGCATACACAGTGGCCTCT	AACAATCCGCCACAAAGACA
mtnr1aa+31k	chr1:17612763-17613299	537	GTGCAACCCATGATTTGAAACA	GGTGAACCCTGCCTTAGACT
pdgfab−8k	chr3:12826209-12826797	589	ACAGACGACAGACAGACAGA	ATGCCCAAACCCCTGATACA
prdm1c+6k	chr20:48644764-48645028	265	AGCATTCTGAGTCATTGAGGC	ACAATTCGTGATGTGTGAAAACC
prdm5+120k	chr23:1908062-1908966	905	TGTTATTCTCTCTATCCTGCCACCA	TTCAACATTTCAGCTGGCCC
ptk2aa+9k	chr19:1853609-1854509	901	AGTTTCTTATCCCCTCCCCA	GTGTGTGTGTGTCTGTGTGT
rasl11a−13k	chr7:51058598-51059197	600	TTGACAACTACATGCAGCAAGA	TTAATTGCACAGTCCTAGATTGC
rmb7−120kb	chr18:47186842-47187247	406	GAGCACAAGGACAGCATAGC	ACTATTGTTGACATGCTGTTCGT
runx1−102k	chr1:1356289-1356844	556	GCCTCTACACTCATTCTGGC	TCTGTGTGTGCAAGTGTGTG
swap70b+3k	chr18:16924893-16925515	623	TTTGTGAAGTGGGTCTGTGC	GATTTCACAATGCACCACAGC
Negative controls				
atp5g3a−14k	chr9:2322648-2323311	664	TGTGAGGGGACAGTGCTAAC	GGGTCATTTCGGATACAGCC
lmbr1+11k (ZRS)	chr7:40398267-40399390	1124	ATCGCACACCACAGATGTTG	CACAAGCAGGCCATTTGACT
lnpa+56k	chr9:2075585-2076205	621	CCGATCGCCCACTATTAGCA	TAAAAGCGGTGAATCGGGTG
six3b−36k	chr12:25473077-25474565	1489	GGGGTACCCGCCATCTCTGAAAACATCA	ACCGCTCGAGGACAACAGGTCCATGTGTGG
six3b−39k	chr12:25470570-25471318	749	GAGGCAGGGGGTAAGTTTTC	CAAAACGCTAACCAAGCAAA
six3b−102k	chr12:25406188-25406890	703	TTCATTCGCTCATTCATTTCC	CGATGATGACGACGACAACT
six3b−104k	chr12:25404546-25405914	1369	GGGGTACCGCACCTGTGCTTTCACTCAA	TGCGAGCTCACCATCCACACGTTACAGC
wnt3−11k	chr12:22125867-22126566	700	TATGCGGCTGTGGTGATAAG	ATGGCTCACGTTTCAATTCC
zgc:173726−2kb	chr22:2473460-2473891	432	TAACTTCAGGCCACCGTTTG	TAGTGGTGGGCAAAGCGAG

c

Fra1 target genes	Covered exons	Length	Forward PCR primer sequence	Reverse PCR primer sequence
runx1	exons 3 and 4	137	GTGGCTCGTTTCAATGACCT	GGTCCGTCCACTGTGATCTT
fgf20a	exons 1, 2 , and 3	148	GAGGAAGGACCACAGCAGAT	ACACTCGGCTGACAGCTTTT
ak5l	exons 1, 2 , and 3	198	GAGAGCATGTTAACAGGACTGATG	ACCACCATCGAGTTTGCAG
dachc	exons 2 and 3	95	GAATGTGACATCACCCGACA	CAGCTGTTAGGCCAGTGTGA
tph1b	exons 4 and 5	153	TACTGAGGTGCCTTGGTTCC	ATGGCAAGATCCGCAAAATA
inhbab	exons 1 and 2	80	CAGCCCTTCGAGATCATCA	CTGCCCTCCTTGGAAATGT
Non target genes				
actl6a	exons 4 and 5	103	TCAGAGGCCTCGTGGAAC	GCACAGCTGATTTACACAAGAA
ctsla	exons 6 and 7	113	TGAGAAGGAGTGCAGCAGTG	TCCAGCTGTTTTTGACAATCC
mmp13b	exons 2 and 3	100	CAAACGTGACCTTCAGGATTC	GTCACTTTGCTCCAGACGTTC
Internal control gene				
actb2	exons 3 and 4	74	CAACAGGGAAAAGATGACACAGAT	CAGCCTGGATGGCAACGT

Table S4 Primer sequences used in this study. **a** Primers used for ZEDtw vector generation. **b** Primers used for enhancer element cloning. **c** Primers used for qRT-PCR.
