# Supplementary material for: Regenerating zebrafish fin epigenome is characterized by stable lineage-specific DNA methylation and dynamic chromatin accessibility
Source: Genome Biol. 2020 Feb 27;21:52. doi: 10.1186/s13059-020-1948-0 (PMC7047409; doi:10.1186/s13059-020-1948-0)
Supplement: Supplementary file 5 — Review history. [file 13059_2020_1948_MOESM5_ESM.docx]

Review History

**First round of review**

**Reviewer 1**

**Comments to author:**

In this manuscript, Lee et al performed whole-genome bisulfite sequencing, ATAC-Seq, and RNA-Seq in two zebrafish cell types, before and after injury. The authors observed that, despite large changes in gene expression, DNA methylation changes were minimal. In contrast, changes in open chromatin were much more abundant. The authors define a resource of open chromatin changes, predicted validated regeneration-specific enhancers, and validated these using in vivo reporter assays. Using a transcriptional regulatory network, they then predicted and tested regulatory molecules driving regeneration.

Overall, this is a carefully performed study to examine the transcriptional and epigenetic changes during regeneration in two tissue systems. Generally, the conclusions are well-supported by the data. However, the overall genomic conclusion that changes in RNA expression correlate more with changes in open chromatin instead of DNA methylation are not surprising. That said, the system where these analyses performed is novel, and the authors go beyond typical descriptive analyses to perform in vivo reporter assays and perturbation experiments. These aspects add to the novelty of this study.

The only analysis I found less than convincing is Figure 5f. First, adding whiskers to the box plots would be informative. Second, it would be informative to highlight a few examples of genes that are differentially repressed in fols1a mutant fish compared to wild-type fish. Validation by a non-sequencing approach like qPCR would be more informative, as an independent means of detection.

Minor comments:
1.     The manuscript has numerous grammatical errors.

**Reviewer 2**

**Comments to author:**

The manuscript by Lee and colleagues describes detailed epigenome and transcriptome maps of zebrafish fin regeneration.  The manuscript is well-written and will likely provide a useful resource for those interested in the topic of molecular mechanisms of vertebrate limb/fin regeneration. Nevertheless, it is fair to say that none of the findings reported come across as particularly surprising. My suggestions for improvement are outlined below:

1) The title is misleading. DNA methylation displays very little dynamics during this process and the fact that lineage-specific methyl-marks are not remodelled during regeneration is the least interesting aspect of the study. I find the identification of novel regeneration-specific enhancers and the fos1a phenotype the most interesting points.

2) The authors refer throughout the manuscript that the identified DMRs are likely just false positives. If so, these panels should be either removed or the data re-analysed to demonstrate whether this is indeed the case. While this study is restrained to a very specific experimental system, there are plenty of published zebrafish datasets (ChIP, ATAC, BioCAP etc) that might provide further insight into the molecular makeup of these DMRs.

3) The first two figures can probably be made supplementary or merged into one as they add very little to the story.

4) The authors comment on how previous studies suggested that DNA demethylation of active cis-regulatory elements is often correlated with gain of chromatin accessibility, a feature that they did not observe in their experiments. With that in mind, it would be useful to comment on how the investigated cis-regulatory elements differ from the ones previously tested (difference in CpG density, motifs). Is this a general feature of the zebrafish regulatory landscape or just something specific to these enhancers?

5) It is not clear how the network in Fig 5b is constructed - this requires further explanation.

**Authors Response**

**Point-by-point responses to the reviewers’ comments:**

Reviewer #1:

In this manuscript, Lee et al performed whole-genome bisulfite sequencing, ATAC-Seq, and RNA-Seq in two zebrafish cell types, before and after injury. The authors observed that, despite large changes in gene expression, DNA methylation changes were minimal. In contrast, changes in open chromatin were much more abundant. The authors define a resource of open chromatin changes, predicted validated regeneration-specific enhancers, and validated these using in vivo reporter assays. Using a transcriptional regulatory network, they then predicted and tested regulatory molecules driving regeneration. Overall, this is a carefully performed study to examine the transcriptional and epigenetic changes during regeneration in two tissue systems. Generally, the conclusions are well-supported by the data. However, the overall genomic conclusion that changes in RNA expression correlate more with changes in open chromatin instead of DNA methylation are not surprising. That said, the system where these analyses performed is novel, and the authors go beyond typical descriptive analyses to perform in vivo reporter assays and perturbation experiments. These aspects add to the novelty of this study.

(1.0) *We thank the reviewer for the encouraging comments on the performance and the novelty of our manuscript. We believe that our analyses and rigorous validation experiments on this regeneration system provide a valuable and novel resource to the science community of the regenerative medicine.*

The only analysis I found less than convincing is Figure 5f. First, adding whiskers to the box plots would be informative. Second, it would be informative to highlight a few examples of genes that are differentially repressed in fols1a mutant fish compared to wild-type fish. Validation by a non-sequencing approach like qPCR would be more informative, as an independent means of detection.

(1.1) *We thank the reviewer for the constructive suggestions. In our revised manuscript, we added whiskers, notches, and individual data points on the box plots in the new Fig. 4f. We also highlighted several example genes whose upregulation were affected by the fosl1a mutation (Additional File 1: Figure S6i). We further performed qRT-PCR and validated that these genes were indeed expressed in significantly lower levels in the fosl1a mutant zebrafish than in their wildtype littermates. We have added this validation in the revised manuscript and have included here for the reviewer’s reference.*

Minor comments:

1. The manuscript has numerous grammatical errors.

*(1.2) We apologize for our grammar errors. We consulted with native English speakers and corrected many grammar errors in our revised manuscript. Those corrections can be found and highlighted in but not limited to the lines 28, 30, 31, and 39. Please note that not all of the grammar corrections were highlighted in the revised manuscript for the ease of reading.*

Reviewer #2:

The manuscript by Lee and colleagues describes detailed epigenome and transcriptome maps of zebrafish fin regeneration. The manuscript is well-written and will likely provide a useful resource for those interested in the topic of molecular mechanisms of vertebrate limb/fin regeneration. Nevertheless, it is fair to say that none of the findings reported come across as particularly surprising. My suggestions for improvement are outlined below:

(2.0) *We thank the reviewer for the compliments on our manuscript. We agree that our manuscript will serve as a useful resource for the community interested in vertebrate regeneration.*

1) The title is misleading. DNA methylation displays very little dynamics during this process and the fact that lineage-specific methyl-marks are not remodelled during regeneration is the least interesting aspect of the study. I find the identification of novel regeneration-specific enhancers and the fos1a phenotype the most interesting points.

*(2.1) We thank the reviewer for the suggestion. We agree with the reviewer that identification and validation of regeneration-specific enhancers and putative upstream transcription factor are ones among the most interesting aspects of the study. At the same time, we also believe that the discovery of the stable DNA methylation signature during regeneration is unexpected and intriguing, especially in the context of maintenance of cellular identity while undergoing drastic cellular morphology changes during regeneration. This surprising finding shed lights on potential mechanisms with which cell fate is restricted during zebrafish fin regeneration. However, we agree that the title in the original manuscript can be misleading by highlighting only one epigenetic mark – DNA methylation. In the revised manuscript, we followed the reviewer’s suggestion and changed the title into “Regenerating zebrafish fin epigenome is characterized by stable lineage-specific DNA methylation and dynamic chromatin accessibility.”*

2) The authors refer throughout the manuscript that the identified DMRs are likely just false positives. If so, these panels should be either removed or the data re-analysed to demonstrate whether this is indeed the case. While this study is restrained to a very specific experimental system, there are plenty of published zebrafish datasets (ChIP, ATAC, BioCAP etc) that might provide further insight into the molecular makeup of these DMRs.

*(2.2) We thank the reviewer for giving us the opportunity to clarify this important point. Here, false positive DMR is a statistical concept. We first established an empirical false discovery rate by calculating DMRs between biological replicates at genome-wide scale. This number is on the order of hundreds. These DMRs usually reflect biological variations or stochastic events, and could be used to represent expected number of DMRs we could identify just by chance. When we calculate DMRs between two tissue types or biological states, we use this expected number to estimate false discovery rate. For example, if we identified 1,000 DMRs between two cell types, while expecting 100 by chance, then the estimated false discovery rate would be 10%. Statistically, this means that 900 of the 1,000 DMRs are likely true positives, and 100 are false positives. Importantly, this is a statistical concept, which means that we only know there are about 100 false positives, but we do not know which 100 are false positives. In contrast, if we identified 200 DMRs between two cell types while expecting 100 by chance, the false discovery rate could be as high as 50%. This means at least half of the DMRs can be expected by chance, however we do not know which half purely based on the statistics. Thus, the overall statistical confidence for these DMRs is low. Note that we estimated the false discovery rate at different statistical cutoffs as well as using two different computational tools to identify DMRs (Additional File 1: Figures S1g, h and S2e, f), and the results were quite consistent.*

*When we attempted to identify regeneration-specific DMRs by comparing cells from pre-injury fins (0 dpa) and regenerating fins (4 dpa), the number of DMRs is also on the order of hundreds, similar to the baseline established by comparing biological replicates. Thus, the number of DMRs discovered is similar to the number of DMRs expected by chance, and the estimated false discovery rate is high. Therefore, although they appear to be real DMRs based on DNA methylation data, they might reflect biological variation or stochasticity, and we do not have enough statistical confidence to determine them as regeneration-specific. We have now further edited the text to clarify why we suggest that regeneration DMRs to be most likely false positives.*

*The reviewer’s suggestion to characterize these genomic regions by using publicly available zebrafish genomic data is an excellent one. True DMRs likely enrich for regulatory elements more so than DMRs representing biological variation. Previous studies have mapped regulatory elements associated with zebrafish development. By taking the reviewer’s suggestion, we used the publicly available datasets [1, 2] to ask whether these genomic regions (regeneration DMRs) have any molecular features. We included the false positive DMRs detected between two biological replicates to compare with regeneration DMRs. We found that the both false positive DMRs and regeneration DMRs are slightly enriched in promoters (Response Fig. 2a) and have high CpG density (Response Fig. 2b). Both sets of these genomic regions maintain high methylation levels during the development and very low signals of histone marks for regulatory elements (Response Fig. 2c). This indicate that these regeneration DMRs have similar molecular features with the real false positive DMRs, at least during zebrafish development.*

*In summary, we believe these data are consistent with our interpretation: true regeneration-specific DMRs are rare if they exist at all, and cell type-specific DNA methylation is in general stable during regeneration.*

3) The first two figures can probably be made supplementary or merged into one as they add very little to the story.

*(2.3) We thank the reviewer for the suggestion. While we believe that the stable lineage-specific DNA methylation signature during regeneration is an unexpected discovery as we discussed above (Response 2.1), we followed the reviewer’s suggestion. In the revised manuscript, we moved the Figure 1 of the original manuscript to the Additional File 1 and combined into the new Figure S1.*

4) The authors comment on how previous studies suggested that DNA demethylation of active cisregulatory elements is often correlated with gain of chromatin accessibility, a feature that they did not observe in their experiments. With that in mind, it would be useful to comment on how the investigated cis-regulatory elements differ from the ones previously tested (difference in CpG density, motifs). Is this a general feature of the zebrafish regulatory landscape or just something specific to these enhancers?

*(2.4) This is a very insightful comment! A direct comparison between cis-regulatory elements we identified in this study and previously characterized cis-regulatory elements is the most effective way to reveal similarities and differences that reflect mechanisms of action. Therefore, we compared the cisregulatory elements (Regeneration DARs) we reported in our study and the zebrafish developmental enhancers identified from the previous study (Phylo(−)DMRs) [2]. Phylo(−)DMRs showed demethylation and gain of active enhancer histone marks during phylotypic period of zebrafish development, while our regeneration DARs were preset as and remained hypomethylated during regeneration (Fig. 3c in the revised manuscript). We found that only 0.96% of DARs were overlapped with Phlyo(−)DMRs, indicating that the two sets are distinct cis-regulatory elements. We compared genome distribution, CpG densities, DNA methylation and chromatin marks during development [1, 2], and enriched motifs in these two sets of cis-regulatory elements. We found that these two sets of cis-regulatory elements have indeed different characteristics. In the revised manuscript, we added this comparison as new Figure S5 and have included here for the reviewer’s reference.*

5) It is not clear how the network in Fig 5b is constructed - this requires further explanation.

*(2.5) We apologize for the lack of clear description of our methods. In the revised manuscript, we added more detailed steps on how the regulatory network was constructed in the Methods section. Here we included the revised paragraph describing how we constructed a putative regulatory network for the reviewer’s reference.*

*Page 24: “To build putative regulatory network, TFs were linked to their downstream target genes, with a similar method as the one described previously [3]. First, TFs whose motifs were enriched in DARs that gained accessibility during regeneration either in sp7+ or in sp7− cells were chosen by HOMER as described above. Then, the expression levels of the zebrafish ortholog genes corresponding to those TFs were examined. Among them, TFs whose expression levels were higher in regenerates at 1, 2, or 4 dpa than in uninjured fins were further selected as putative upstream factors. Their new bound sites in fin regenerates at 4 dpa were identified by intersecting TF-bound sites predicted by CENTIPEDE and DARs that gained accessibility during regeneration. Finally, TFs with those new bound sites in fin regenerates at 4 dpa were chosen to build putative regulatory network. The closest TSS from each new bound site was identified by BEDTools and it was classified as a putative downstream target gene of a given TF if that closest gene was upregulated during regeneration. Putative TF-TF regulations and TF auto-regulation were also identified in case that a putative target gene was one of the chosen TF. The enriched GO terms from Metascape analysis were used to annotate the putative downstream target genes.”*

RESPONSE REFERENCES

1. Bogdanovic O, Fernandez-Miñán A, Tena JJ, de la Calle-Mustienes E, Hidalgo C, van Kruysbergen I, et al. Dynamics of enhancer chromatin signatures mark the transition from pluripotency to cell specification during embryogenesis. Genome Res. 2012;22:2043-53
2. Bogdanović O, Smits AH, de la Calle Mustienes E, Tena JJ, Ford E, Williams R, et al. Active DNA demethylation at enhancers during the vertebrate phylotypic period. Nat Genet. 2016;48:417-26.
3. Lee HJ, Lowdon RF, Maricque B, Zhang B, Stevens M, Li D, et al. Developmental enhancers revealed by extensive DNA methylome maps of zebrafish early embryos. Nat Commun. 2015;6:6315.

**Second round of review**

**Reviewer 2**

The authors have addressed all of my concerns. They have provided detailed analyses of DMR regulatory signatures and explained how those compare to previously published data. Other minor concerns / comments were also successfully addressed.
